# Fading boundaries between the physical and the social world: Insights and novel techniques from the intersection of these two fields

**DOI:** 10.3389/fpsyg.2022.1028150

**Published:** 2023-02-13

**Authors:** Cecilia Dapor, Irene Sperandio, Federica Meconi

**Affiliations:** Department of Psychology and Cognitive Science, University of Trento, Rovereto, Italy

**Keywords:** social cognition, visual perception, embodiment, body indexes, posture, empathy, social body

## Abstract

This review focuses on the subtle interactions between sensory input and social cognition in visual perception. We suggest that body indices, such as gait and posture, can mediate such interactions. Recent trends in cognitive research are trying to overcome approaches that define perception as stimulus-centered and are pointing toward a more embodied agent-dependent perspective. According to this view, perception is a constructive process in which sensory inputs and motivational systems contribute to building an image of the external world. A key notion emerging from new theories on perception is that the body plays a critical role in shaping our perception. Depending on our arm’s length, height and capacity of movement, we create our own image of the world based on a continuous compromise between sensory inputs and expected behavior. We use our bodies as natural “rulers” to measure both the physical and the social world around us. We point out the necessity of an integrative approach in cognitive research that takes into account the interplay between social and perceptual dimensions. To this end, we review long-established and novel techniques aimed at measuring bodily states and movements, and their perception, with the assumption that only by combining the study of visual perception and social cognition can we deepen our understanding of both fields.

## 1. General introduction

New insights on how traditional neuroscientific methodologies and instruments can adapt to emerging models of social cognition and perception are long needed. The vast majority of studies on perception so far have mainly concentrated on dissecting bottom-up processes, such as feature categorization and grouping. However, higher cognition seems to be rooted in our sensory representation of the world and to be modeled by our physicality. Therefore, we need research instruments able to tap into the deep embedding of our cognition in bodily processes. After all, the interface between the environment, whether physical or social, and its perception at our end comes down to our body.

For instance, this article focuses on the subtle interactions between sensory input and social cognition in visual perception and proposes the body indices, such as gait and posture, as a critical mediator of such interactions.

We structure our perspective in three sections: the first one starts by exposing different approaches in perception studies and by highlighting the deep interplay between top-down and perceptual processes; the second one focuses on embodied sensorial aspects of social cognition and its reliance on visuospatial behavior mechanisms; the third one presents a selection of studies in which bodily states (physiological, postural, and kinematical) are analyzed in their role of conveying social information to the viewer.

The key idea behind this work is that sociality is a part of – and not apart from – our biological being: we have preconscious dispositions toward perceiving social cues, namely other people’s bodies and their expressions, and the way we perceive the (social) environment is shaped by our own body and its expressions. Accumulating evidence from different lines of research is supporting this notion, revealing that our perceptual experience is not a mere copy of the external appearances, but rather the result of a compromise between physical environment’s layouts and people’s social needs and expectations (e.g., Balcetis and Dunning, 2009; Firestone and Scholl, 2016; Niedenthal and Wood, 2019; Sato et al., 2019; Valenti and Firestone, 2019; Sun et al., 2021).

Building upon compelling evidence showing an overlap between spatial and social behavior in the perception of distances (Henderson et al., 2006; Bar-Anan et al., 2007; Yamakawa et al., 2009; Xiao and Bavel, 2012; Parkinson and Wheatley, 2013; Takahashi et al., 2013; Hamilton et al., 2014; Schiano Lomoriello et al., 2018; Sato et al., 2019; Kroczek et al., 2020), and on studies on the preconscious and automatic processing of social visual stimuli (Morris et al., 2001; Pegna et al., 2005; Tamietto and de Gelder, 2008; de Gelder, 2009; de Gelder et al., 2010; Zhou et al., 2019), we claim that in the process of understanding others, social top-down and visual bottom-up processes join their forces to prompt an adaptive response to the social stimulus. Moreover, we will show that the exploration of our physical and social surroundings is self-centered, or, more precisely, body-centered. In other words, our body shape and appearance strongly affect the way we move and conduct ourselves toward physical and social entities (Yee et al., 2009; Linkenauger et al., 2010; van der Hoort et al., 2011), revealing a fundamental role of our body in shaping our perception and our sociality.

In short, the main claim of this review is that our body is a critical mediator of the interplay between social cognition and visual perception; the body is indeed our interface at the basis of our interaction with the environment. We can trace the effects of this mediation even in the rescaling effects that it exerts on our sight: understanding the deep influence of our bodies and bodily processes on our visual perception is a crucial step to frame also its role in shaping our attitude toward and interpretation of social relationships. We observe other people’s postures and movements in order to understand their emotions or intentions, and the effective recognition of the others’ psychological state is accompanied by the instinctive simulation of their expressions in our own body. Theories of embodied cognition (Goldstone and Barsalou, 1998; Niedenthal et al., 2005; Proffitt, 2006; Gallese, 2009) claim that we use sensorimotor models originating from our proprioception and body schema (see definition in Box 2) to infer the psychological state of others. However, the literature often lacks the specification of the exact bodily indexes observed and simulated during social processes. We want to help fill this gap by presenting and promoting novel techniques that integrate the study of social cognition with physiological and visual measures, aiming at incentivizing integrative and multimodal approaches. To sum up, we strongly encourage the re-integration of the whole body in the study of social behavior and visual perception, following Nakayama’s claim that “vision science is going social” (Nakayama, 2011).

## 2. Reconstructing visual perception

The concept of perception has evolved throughout history. The last century has seen different theories succeeding one another in an attempt of giving a systematic explanation of how humans perceive the world surrounding them. Since the first definitions of perceptual processes, a differentiation between early sensory systems and higher cognitive processes has emerged. Although a reciprocal influence between these different levels of processing is well-established in the literature, an unsolved issue concerns the stage at which these two levels start interacting. For instance, traditional views assume a hierarchical structure in which the external input is initially processed by sensory cortices and it is only later ri-elaborated and interpreted by higher cognition. In contrast, alternative approaches (e.g., New Look, enactivism, ecological psychology, 4E approaches) argue for an early intervention of motivational systems, which modulate our perception by increasing the saliency of some objects on the basis of our current needs or desires. Within this other theoretical framework, perceptual processes have a constructive nature for which external information is gathered and modeled for the demands of our inner states (Balcetis and Dunning, 2006).

### 2.1. New look and other perspectives in perception: Visual perception as a constructive process

According to the New Look theory, perception is far from being a neutral and objective representation of the external world. Instead, what we see appears to be the result of a compromise between autochthonous and behavioral determinants (Bruner and Goodman, 1947). While *autochthonous* refers to the electrochemical signals generated by the sensory end organs, *behavioral* includes learning and motivation, social needs and attitudes, basic physiological needs, such as hunger and thirst. These two determinants build a perceptual hypothesis that undergoes a selection process driven by our needs or requirements. Objects that comply with the selective criteria become “more vivid, have greater clarity or greater brightness or greater apparent size” (Bruner and Goodman, 1947). In a classic experiment, Bruner and Goodman (1947) showed that a physical entity, like a metal disk, invested of social value (e.g., a coin) is perceived as having a bigger shape compared to the same form without any utility (i.e., a simple metal disk). The authors postulated that the greater the need for a socially valued object, the more marked the reshaping of the perceived entity would be. In agreement with this hypothesis, they demonstrated that poorer children were more likely to perceive the coins as bigger, compared to an age-matched group of more wealthy individuals (Bruner and Goodman, 1947).

Despite initial criticism, this theory has recently regained attention thanks to scholars who have developed rigorous methodological approaches and demonstrated how semantic knowledge (e.g., stereotypes) can shape - at a very early stage – the way we process sensory inputs (Otten et al., 2017).

Two additional influential theories that date back to the second half of the last century and originated in opposition to cognitivism, are the enactivism and ecological psychology. The latter was pioneered by Gibson (2014) and has its foundations in his perspective on visual perception. The most relevant point of this framework is the concept of affordances (see Box 1), a notion that has been exploited in different fields and transposed from the physical environment of inanimate objects to the social affordances offered by another person. Another fundamental aspect of ecological psychology is the reintroduction of the body as a reference point for our perceptions, and the concept of perception as an act of information pickup. Gibson revolutionized the whole field of visual perception, introducing new models based on the way that our visual sensory system is built on the idiosyncrasies of our body rather than the external stimulus.

While one can easily consider ecological psychology as a new theory of perception, the redefinition of the organism-environment relationship imposed by this theory has important implications for cognition (Popova and Rączaszek-Leonardi, 2020). A more radical elaboration of the centrality of our body in cognitive processes has been put forward by the enactivist approach, largely founded on the ideas of Varela et al. (1991). Similar to ecological psychology, this theory strongly highlights the importance of the relationship between agent and environment, with the assumption that cognition and perception emerge together and are so closely connected that they cannot be considered separately. The objectivity of perception is deconstructed in favor of the inevitability of the subjective state in the global landscape formed by the individual immersed in its surroundings (Fuchs, 2020; Heft, 2020; de Pinedo García, 2020; Popova and Rączaszek-Leonardi, 2020; Read and Szokolszky, 2020).

This account of perception conforms also to the more and more popular theory of predictive coding (PC), which has its origin in the model of visual perception formulated by Rao and Ballard (1999). The authors described visual perception not only as a feedforward loop from lower-to higher-order visual cortical areas, but also as a cycle of prediction and error-correction. The new emerging ideas that followed this initial assertion, are based on the inferential nature of the brain (Friston, 2018). In a nutshell, the PC framework states that our neural networks constantly predict sensorial aspects of the environment based on the statistical regularities of the natural world. This prediction generates a perceptive model which is confronted with the incoming sensory inputs and corrected in case of mismatch (Rao and Ballard, 1999; Friston and Kiebel, 2009). One can easily see the parallelism with Bayesian modeling in statistics, for which an hypothetical or aprioristic model of a certain phenomenon is continuously updated on the basis of the newly collected data. What the system, in this case the brain, should focus its energy on is to minimize the prediction error (Friston and Kiebel, 2009; Friston, 2018). This inferential view of our cognition and sensorium, described also as Baesyan brain hypothesis, has gained growing consensus and has become one of the most dominant models in cognitive neuroscience. The PC approach has been applied in multiple fields of science. A relevant example is its application to explain interoceptive awareness or accuracy (Seth et al., 2012; Ainley et al., 2016). In the study of Ainley et al. (2016), the sensitivity for internal bodily information is explained as the ability to adapt a “prior” representation of the body state to the effective interoceptive sensation, by continuously adjusting and minimizing the prediction errors. If this predictive dynamics holds true for interoceptive inputs as well and not only for what we expect to perceive from the external environment, then the model can be extended to partially explain our emotional responses (Seth, 2013; Barrett and Satpute, 2019). In fact, emotions can be seen as predictions of reactions to an event based on past experiences that generated models of behavior. They can represent the active inference of how to respond to a particular situation, and be created from memory with the possibility of future adjustments. In a more articulated manner, Seth (2013) integrates the interoceptive predictive coding hypothesis in a coherent understanding of emotional content as an “active top-down inference of the causes of interoceptive signals.” Interoception is no longer considered as a one-directional collection of bodily sensations from peripheral receptors to the central processor, but rather as a process shaped by top-down inferences and influences along with bottom-up error-correction processes. In summary, the predictive coding account of emotions defines them as active inferences on the causes of physiological changes, both at internal and external representational levels (Seth, 2013; Barrett and Satpute, 2019).

Again, the “strange inversion” brought about by this theoretical account goes in the same direction as the other frameworks described so far, which point out the inferential, constructive, and active functioning of the perceiving organism, whilst leaving behind the idea of the brain “as a glorious stimulus–response link” (Friston, 2018). Following this discussion, it is probable that the “naive realism,” supported by traditional approaches, whereby what we see, smell and feel is a faithful representation of what is “out there,” will be progressively replaced by a view of our visual system as grounded in action capabilities and social influences (Proffitt and Linkenauger, 2013; Proffitt and Baer, 2020). The current review aims at pushing cognitive research into this direction, highlighting the deep influences that our inner states and social needs exert on perceptual processing. In the ensuing sections, we will argue that the visual salience of an object is given by its affordances (see definition in Box 1) to satisfy our physiological or social needs. In other words, we project on the objects surrounding us different degrees of desiderability – based on our bodily and psychological states - and perceive them accordingly, inevitably bonding our perception to our action possibilities.

**BOX 1 Definition of affordances.**

The term was introduced by James Gibson, within its ecological approach to the study of vision, and indicates the totality of actions that an object allows to perform. The affordances of an object can be defined as the potential actions elicited by the view of that object. For example, seeing an apple suggests the actions of eating it, grabbing it, or moving it. A chair “affords” being sat on, and so forth.

### 2.2. Our metabolic energies influence visual perception

Our action possibilities are delimited by the resources we dispose of and the costs of the actions we want to undertake. Using Proffitt’s words, “survival for any organism, including people, is a matter of resource management” (Gross and Proffitt, 2013). Our brain calculates costs and opportunities associated with every movement, and this constant evaluation is carried out automatically and unconsciously, in that, if we had to be aware of it, our executive functions would overload (Proffitt, 2006; Gross and Proffitt, 2013).

First and foremost, our ability to perform an action depends on our body characteristics: our body size determines what we can reach as well as what we can see (Linkenauger and Proffitt, 2008; Sugovic et al., 2016). The effect of our body mass on perceived distance was assessed in an experiment of Sugovic et al. (2016) in which normal weight, overweight, and obese individuals were asked to estimate a same distance and report their beliefs concerning their body size. They found that perceived distance was mainly affected by physical body weight and that this effect was independent from personal beliefs. Specifically, they found that the heavier the person, the greater the estimation of distance (Sugovic et al., 2016). Also, the physiological state of our body plays a major role in what we can perform and how. Being tired, or out of training, or carrying a weight, are all factors that can diminish our potential to perform actions (Proffitt, 2006; Linkenauger and Proffitt, 2008; Proffitt and Baer, 2020). A reduction of our action possibilities translates into an adjustment of the environmental perception. In fact, it has been shown that perceived distances increase when our energies are scarce and are instead reduced when we are trained and performative (Zadra et al., 2016; Proffitt and Baer, 2020). In the same fashion, Bhalla and Proffitt (1999) showed that the steepness of a hill was overestimated by participants who were asked to carry a backpack, fatigued runners or those in low physical and health conditions when compared to participants in their full forces (e.g., not carrying a backpack or fit and in good health). A further example on how sugar intake and fitness level affect visual estimates was provided by Zadra et al. (2016). The authors asked participants to judge distances after physical exercise. Prior to the physical activity, half of the participants received a carbohydrate supplement, whereas the other half received a placebo. They observed that those who received the energizer rated the extent to be shorter compared to the placebo group. They also found that perceived distance correlated with other measures of fitness, such as blood glucose, heart rate (HR), and caloric expenditure under physical fatigue, further confirming the influence of bioenergetic resources on perceptual processing (Zadra et al., 2016). Interestingly, Changizi and Hall (2001) demonstrated that thirsty people perceive ambiguous visual stimuli as more transparent than non-thirsty subjects, and this may be due to the implicit association of transparency with water.

### 2.3. Tools extend our action possibilities modifying the perception of our surroundings

Beside dimensions and fitness of our body, another factor that can determine our potential to perform actions is the use of tools. In fact, tools can extend our reach to extrapersonal space, including farther objects in our area of manipulability. Objects within our reach are automatically perceived as candidates for potential actions and this implies different perceptual processings (for a review, see Brockmole et al., 2013). Indeed, they are visually scanned in a more attentive way and with a detail-oriented processing style in order to enable appropriate action responses compared to objects we cannot touch or immediately interact with (Brockmole et al., 2013). Hence, holding a tool that increases the area of our possible interaction with the surroundings immediately modifies the visual perception of what would have been beyond our reach. For example, when patients with hemispatial neglect (a neuropsychological condition characterized by reduced awareness of visual stimuli in one side of the visual field, not accompanied by sensorial deficit) limited to the near space were provided with a stick and asked to perform a line bisection task in the far space, the neglect expanded to include the area reachable with the tool (Berti and Frassinetti, 2000). This demonstrates that the artificial extension of our reach remodels peripersonal space and the perception of far and near objects, suggesting once more that our chance of interaction with the environment has deep effects on how we perceive it.

### 2.4. Social baseline theory: Social resources can directly alter our visual perceptions

Not only metabolic energies weigh on the capacity to undertake any action, but also they influence our social relationships. Supportive social networks allow distributing the efforts of any endeavor and protect the individual from potential dangers. It has even been argued that receiving help from other humans is a matter of survival and that the greatest human strength are other humans (Oishi et al., 2013). Indeed, we are born and raised within a social environment that provides for our basic needs until we can take care of ourselves. Even then, we are for the rest of our lives embedded in social networks (Gross and Proffitt, 2013). Interestingly, similarly to what has been observed for metabolic resources, it has been demonstrated that social resources, too, influence our perception of the environment.

The Social Baseline Theory (SBT), formulated by Beckes and Coan (2011), describes interindividual differences in reacting to social support. According to this theoretical account, social support is considered as a default precondition of our actions and determines the baseline from which we calculate the amount of energy available (Beckes and Coan, 2011; Cole et al., 2013; Gross and Proffitt, 2013). Nevertheless, investigations on the role of social resources in perception have been largely neglected in the literature, especially if compared to the wealth of evidence on the role of physiological states (Gross and Proffitt, 2013). However, accumulating evidence indicates that the presence of a significant other acts as an empowering factor when facing difficulties or specific tasks. For example, Doerrfeld et al. (2012) observed that when participants were asked to lift a box they tended to judge its weight as lighter if they knew they would receive help, compared to when they knew they would lift the box without any help. Schnall et al. (2008) replicated Proffitt’s study on slant perception where slant estimates varied as a function of the physical and health conditions of the observer (see subsection 2.2), but this time the social support factor was also considered. Participants could either be accompanied by a friend or imagine the presence of another person (friendly or not). In both cases, social support decreased the perception of the steepness of the hill. Other studies have tried to shed light on the ways in which social inclusion or exclusion impact our sensory systems. These studies revealed a wide range of effects on visual perception. For instance, the feeling of being understood is another factor that appears to influence distance and steepness perception. In an experiment by Oishi et al. (2013), participants were judged by an evaluator with a few adjectives chosen from a list, from which the same participants had previously picked a few words to describe themselves. In the understanding condition, participants received the same judgments they also had chosen, while in the misunderstanding condition the evaluator judged the participants with words that largely differed from the participants’ self-assessment. After the evaluation session, participants performed a slant estimation task similar to the one used in Bhalla and Proffitt’s study (1999). Results showed that feeling understood decreased the slope estimates compared to the misunderstanding condition (Oishi et al., 2013).

To conclude, we can claim that vision assists our possibility of action by modulating perceived size and distance of objects. Our potential to perform actions, in turn, is determined by our dispositional bioenergetic and social resources. Therefore, we see the environment according to the dispositions of our bodily states and of our social network.

Table 1 provides a summary of the studies cited in this section.

**BOX 2 Definitions of proprioception and body schema.**

Proprioception is the capacity of perceiving and detecting the position of our own body in space, as well as the state of activation of our muscles, without the support of our sight. The collection of combined signals from sensory receptors in the muscle, skin, and joints, allows us to be aware of our limbs position and movements, and it is - in this sense - a fundamental aspect of motor control. In fact, proprioceptive information is integrated in our body schema, which combines the peripheral inputs with central (brain) processes in order to lead the execution of any action or movement. Body schema has been defined as the body representation for action. Indeed, it can extend to incorporate any tool we are holding allowing the sophistication of human tool-use abilities

**Table 1:** Summary table of the studies cited in section 2.

Study	Sample	Stimuli	Design	Results
Sun et al. (2021)	*N* = 18 (13 females, mean age = 22.9)	Target rectangles placed of the top of different stimuli	The different stimuli were selected to induce positive (squirrel), negative (rats), or neutral (wooden blocks) emotions. Participants were asked to rate distances from and size of the targets	Participants perceived the target object on top of the toy rats (induced negative stimulus) significantly closer and larger than the same target object resting on top of the toy squirrels (induced positive stimulus) suggesting that there is a perceptual bias even within reachable personal space
Balcetis and Dunning (2009)	*Experiment 1: N* = 63	Ambiguous figures	Taste-testing of a desirable and a non-desirable beverage. The choice of the beverage/food was based on seeing one of the two possible interpretations of the ambiguous stimulus	Participants’ desire to obtain the desirable reward influenced their interpretation of the ambiguous figure
	*Experiment 2: N* = 43	*Experiment 1:* Number-letter figure, B / 13.		
		*Experiment 2*: Seal-horse figure		
Balcetis and Dunning (2009)	*Experiment 1*: *N* = 90	Desirable objects*Experiment 1*: A bottle of water	*Experiment 1.* Participants were given either a salty snack or a glass of water and then were asked to estimate the distance between them and a bottle of water	Perceptions of distance depend in part on the desirability of the perceived object—which depends, in turn, on its capacity to satisfy a visceral or intrapsychic need
	*Experiment 2a*: *N* = 123	*Experiment 2a*: A 100 dollar bill	*Experiment 2a*. Participants were offered the chance of winning a 100 dollar bill in a card game or only a candy and then asked to estimate the distance from the bill	
	*Experiment 2b: N* = 89	*Experiment 2b:* A survey containing self-relevant feedback	*Experiment 2b.* A survey on sense of humour was ostensibly graded by the experimenter and participants were given either positive or negative feedback and the survey was then placed away from the person and asked to judge how far away	
Zadra et al. (2016)	*N* = 8 (3 females, mean age 26.38)	Walkable distances	A host of physiological measures were recorded as participants engaged in exercise on 2 occasions: once while provided with a carbohydrate supplement and once with a placebo. Distance estimates were made before and after exercise on both occasions	The carbohydrate manipulation caused decreased distance estimates relative to the placebo condition. Individual differences in physiological measures that are associated with physical fitness predicted distance estimates both before and after the experimental manipulations
Bruner and Goodman (1947)	*N* = 30 (mean age = 10)	Disk of different sizes. The disks could either be neutral metal items or coins of different value	The children were asked to estimate the size of the metal disk/coin by adjusting the diameter of a circle of light projected on a screen	Coin size was overestimated, while the neutral disk size estimates were closer to reality. The higher the coin value, the bigger the overestimation. Poorer children overestimated more than more wealthy children the size of the coins
Changizi and Hall (2001)	*N* = 74	Visual stimuli “definitely,” “ambiguously,” or “definitely not” transparent	Participants had to judge the stimulus as “transparent” or “not transparent” in two conditions: after having eaten a bag of chips (thirsty group) or after having drunk water (non-thirsty group)	The thirsty group showed a greater inclination to judge as transparent the ambiguous stimuli
Bhalla and Proffitt (1999)	*Experiment 1. N* = 130 (65 females)	Slant of a hill	Participants were asked to judge how steep the hill was in three ways: verbally, visually, and haptically	All the experiments demonstrated an effect of load, fatigue, or fitness on the slant estimates, but only for verbal and visual assessments
			*Experiment 1.* Participants performed the task in two conditions: wearing a backpack and without it.	*Experiment 1.* Wearing the backpack influenced the slant estimate making the hill look steeper.
	*Experiment 2*. *N* = 40 (20 females)		*Experiment 2.* Participants had to give slant estimates of a hill before a long run (45 to 75 min) and of another hill after the run	*Experiment 2.* The hill estimates were higher after the exhausting run
	*Experiment 3*. *N* = 74 (35 females)		*Experiment 3.* Different fitness measures were taken from the participants (heart rate in different conditions and body mass index) after or before asking them to judge the slant of the hill	*Experiment 3.* A higher level of fitness corresponded to a lesser overestimation of the hill’s slant
Sugovic et al. (2016)	*N* = 66 (30 female, mean age = 24.4)	A cone presented on the sidewalk (4 target distances)	After making four distance estimates, participants completed a survey. Along with demographic questions, the survey asked them to indicate their height, weight, and an evaluative measure of body size	A person’s body weight influenced perceived distance: Those who weighed more than others perceived distances to be farther
Studies involving social manipulation				
Doerrfeld et al. (2012)	*N* = 43 (mean age = 22.5)	Box weight	Participants were asked to judge the weight of a box filled with potatoes in two conditions: alone or with someone else	Participants in the joint condition judged the weight as lighter than in the solo condition
Harber et al. (2011)	*Experiment 1*. *N* = 107 (63% female; mean age = 20.8)	*Experiment 1.* Distance estimates at 3 measurements points	*Experiment 1*. Stimuli were either a live tarantula or a cat toy. Participants were primed with positive, negative or neutral self-worth conditions	Resources moderate the perception of physical threats (dangerous animals, hazardous heights), and are not limited to the implicit calculus of metabolic costs (i.e., how much physical effort a situation might demand, relative to one’s physical resources)
	*Experiment 2. N =* 91 (64.9% female; mean age = 20.18)	*Experiment 2.* Height estimates from the fourth floor of a building	*Experiment 2.* Participants were left free to hold on the handrail or were denied this possibility by tying their hands behind their back. Self-esteem was manipulated	
Cole et al. (2013)	*N* = 48 (100% females)	A male confederate	Participants were shown a video of the male confederate in which he would appear threatening, disgusting or neutral. Then they were asked for distance estimates	Experimentally induced social signals of threat (but not disgust) led to perceived proximity
Oishi et al. (2013)	*N* = 202 (112 female; undergraduate students)	Pain endurance (hand in icy water), slant perception, distance perception	Feelings of understanding or misunderstanding were induced in the participants by judging them with a list of adjectives (positive or negative)	Participants in the understanding condition were able to put their hands in icy water for a longer period of time, perceived the target locations to be closer, and perceived the same hill to be less steep than those in the misunderstanding condition
Schnall et al. (2008)	*N* = 34 (19 female; mean age = 19.94 years)	Slant of a hill	Participants judged the hill slant verbally, visually, and haptically in different conditions: alone or with a friend	Participants with a friend, compared to those alone, saw the hill as less steep. The longer participants knew their friends, the less steep they estimated the hill to be, on both the verbal and visual measures

## 3. Perceiving the physical and the social world

After having grounded our discussion in a constructive and embodied perspective of visual perception, we can now focus on its integration in models of social cognition. We will start by drawing a parallel between the research approaches used in visual perception and those used in social cognition. In both cases, we will conclude that adopting a multimodal integrative perspective can better represent the intertwined and complex nature of these processes. We will then present the embodied account of social cognition, which we believe to be the most accurate explanation of how we understand one another. Finally, we will provide empirical evidence for a common mechanism for mapping social and physical distances, a further confirmation of the tight link between social cognition and visuospatial perception mediated by the processing of body indices.

### 3.1. A parallel between approaches in perception and social cognition

The issue of how we represent objects and events in our mind is and has always been a central theme in cognitive sciences and for a long time these representations have been described as symbolic and amodal. In the field of perception, for example, the main assumption was that we construct abstract representations of the external inputs through mechanisms of feature extrapolation and categorization (Lindblom, 2020). Such a view takes inspiration from Fodor’s modularity, according to which encapsulated perceptual modules in the brain transmit the sensory information to higher processing levels that manipulate them in the form of symbolic representation (Fodor, 1983). The same form of representation - amodal and disembodied - has also been used in the study of social cognition. According to traditional accounts, people process social information by means of categories, schemata, feature lists, semantic networks, and so forth (Landau et al., 2010). However, despite their clarity and linearity, these theories do not account for the multimodal nature of perceptual and social experiences, in which high-and low-level cognitive processes strongly interact (Zaki and Ochsner, 2012). More recently, models in which perception and cognition behave as coupled systems are gaining new ground. In the same way, alternative paradigms of social cognition stemming from theories of embodied cognition (Goldstone and Barsalou, 1998; Niedenthal et al., 2005; Gallese, 2009) are challenging the idea of amodal representations of the social information. We endorse the adoption of multimodal and integrative models of both perception and social cognition, confident that without acknowledging a common basis for perceptual and conceptual processing of physical and social events, our understanding of the brain and the mind would remain incomplete.

### 3.2. An embodied account of social cognition

Empathy is the ability to understand others’ inner state by explicitly inferring it from available contextual information or by internally simulating it (Zaki and Ochsner, 2012; Sessa et al., 2014; Meconi et al., 2018). According to the embodied account of social cognition, we understand other people’s mental state by reproducing it in ourselves (e.g., Niedenthal et al., 2005). This is achieved by internally mimicking the same sensorimotor patterns observed in others, which recall specific psychological states we experienced in association with that physical expression (Gallese, 2007, 2009). This has already been shown in a study by Duclos et al. (1989) where participants were asked to mimic some negative emotion expressions (fear, sadness, anger) by contracting specific muscles. In a first experiment, the expressions were limited to the face, while a second testing involved a full body simulation. Participants were convinced that the study regarded brain lateralization and that the muscles’ contraction was a conflicting task, the function of which was to overload the cognitive system. Finally, they had to report their feelings throughout the experiments, choosing among different emotions and rating their intensity. Although participants were naïve to the aims of the experiment, they reported higher intensity for the emotions they were mimicking in that moment, both for facial and full body expressions, giving strength to the idea that the activation of specific sensorimotor schemas elicits those embodied feelings.

The discovery of the mirror neurons is a fundamental step at the basis of embodied social cognition because mirror neurons are considered as one route to the development of our ability to understand others’ actions. Human mirror neurons seem to be widely spread across the brain with peaks of concentration in the premotor and somatosensory cortices (Gallese, 2007; Fabbri-Destro and Rizzolatti, 2008; Bastiaansen et al., 2009; Gallese, 2009; Keysers et al., 2010; Mukamel et al., 2010). The peculiarity of these neurons lies in the fact that they fire not only when we perform a specific action, but also when we see that same action performed by someone else. Traditionally, research on human mirror neurons adopted fMRI investigations to identify which areas become more active during the observation of another person, and this has allowed the mapping of the neural circuitries that exhibit mirroring properties. Recently, Paradiso et al. (2021) reviewed the scientific production across species to reveal which brain areas are involved in empathic reaction. Empathy is the ability to resonate with the others’ inner state and to explicitly understand it (often referred to as affective and cognitive empathy, respectively). Numerous studies on animal models showed converging results: the anterior cingulate cortex and the amygdala resulted as the main areas involved in empathy-related phenomena. The same authors also reviewed the literature on the role of analgesics in modulating prosocial behavior which shows that reducing pain perception hinders the ability to empathize with the pain of others. In fact, the most recent trends in research on empathy focus on the mechanisms of empathy for pain (Rütgen et al., 2015, 2018; Lamm et al., 2019). This new research direction sought to provide mechanistic explanations to simulation models, by selectively disrupting specific subprocesses - nociception in this case - with different techniques (e.g., tDCS, analgesics) to verify their involvement in cognitive processes, like empathy for pain (Bonini et al., 2022; Maggio et al., 2022). The empathic experience of others’ pain has been widely examined by Rütgen and Lamm, who conducted several studies on the role of our own nociception in the ability to recognize and understand others’ pain. In an fMRI experiment of 2015, the researchers manipulated participants’ nociception (i.e., the encoding of noxious stimuli) by means of placebo analgesia induced in half of the participants. FMRI data was collected, while a painful electrical stimulation was delivered either to the participant or to another person present in the scanner room. Results showed reduced activation of the anterior insular and midcingulate cortex, areas typically involved in empathic responses for pain, in the group of participants in which placebo analgesia was administered compared to the control group in which participants did not receive any treatment. Along with other evidence (Rütgen et al., 2015, 2018), these findings are in line with those studies showing that incidental (Forkmann et al., 2015) and voluntary (Fairhurst et al., 2012) reinstatement of an autobiographical pain, involves the partial recruitment of the brain areas that encoded nociceptive stimuli at the time of memory formation. Indeed, memories of autobiographical physical pain augment participants’ cognitive empathy for other individuals depicted in similar physically painful situations (Meconi et al., 2021).

Neuromodulation and lesion studies are also suited to pinpoint the networks underlying mechanisms of embodied social cognition. Such an example is the experiment of Lenzoni et al. (2020), which demonstrated a causal relationship between body expression and emotion recognition. Using a matching task of faces and bodies, the authors measured social abilities in patients with myotonic dystrophy, a neuromuscular disease which induces strong sensorimotor limitations. The clinical population performed significantly worse than the group of healthy controls, demonstrating a causal role of visuomotor abilities in emotion recognition. In a review by Keysers et al. (2018), neuromodulation and lesion studies were presented as evidence for the fundamental role of the primary somatosensory cortex and mirror neurons network (parieto-premotor areas) in understanding and predicting the actions of others, which is in turn connected with the ability of recognizing their emotions.

The relevance of bodily states in our social judgments is also rooted in our language. For example, feelings of affection and love are usually described as warm, such as the experience of a hug, while loneliness and social distance are typically associated with cold attributes (e.g., “giving someone a cold shower,” “cold-hearted”). Ijzerman and Semin (2009) provided evidence for this deep interdependence between language, perception and social behavior. The authors prompted different temperature conditions by asking their participants to rate the social proximity they felt with a known person of their choice while they were holding cold or hot beverages. As it turned out, the warm condition was associated with greater social proximity, compared to the condition of holding a cold beverage.

Taken as a whole, these findings suggest that bodily experiences can play a preconscious and automatic role in shaping explicit awareness and in leading our interaction with the world. We can even state that without embodying our own and others’ psychological states, we are denied the possibility of understanding them. Such a conclusion leads again to the necessity of adopting an integrative approach for studying both perceptual and cognitive mechanisms. In the next subsection, we provide more evidence for the reliance of cognitive processes on perceptual ones, by showing that we recruit the same neural networks dedicated to visuospatial representations of distances to represent different degrees of social proximity.

### 3.3. When the social meets the spatial: Interpersonal distances

A large body of literature highlights that we use overlapping systems for assessing social proximity and physical distances. For instance, Bar-Anan et al. (2007) used a Stroop-like task in which words indicating close or distant social affiliation (“us” or “enemy”) were positioned in closer or farther perspectives. Participants had to indicate if the item’s position on the screen was proximal or distal, independently from the meaning of the word. It resulted that words were classified faster when the psychological and the spatial distances were matching, compared to when the two types of distance were incongruent. For example, when the word “us” was written in a close-up position in the scenario, the response time was shorter compared to the condition in which the same word (indicating social proximity) was positioned in the background of the scenario. The authors interpreted this finding in terms of a common mechanism for the processing of spatial and psychological distances (see definition in Box 3), which would explain the slower response in the incoherent condition due to the activation of incongruent representations on the same neural path.

Another important line of research supporting this view is the one that investigates the interpersonal distance in social interaction. It is commonly known that we adjust our position in relation to our intimacy with the people around us (Hall, 1963; Hall et al., 1968; Lenzoni et al., 2020). This effect has been named and described in multiple ways. For instance, Teneggi et al. (2013) defined peripersonal space as a multisensory-motor interface between body and environment and showed that its shrinkage or extension depended on the presence and interaction with others. In this vein, Serino (2019) extensively reviewed the literature on peripersonal space, highlighting the stretchable nature of this multisensorial space and its role in mediating body-environment interactions. Furthermore, the author claimed that this physiological construct has the psychological consequence of defining the boundaries between ourselves and the external world, enabling bodily self-location and consciousness (Serino et al., 2013; Blanke et al., 2015; Noel et al., 2018). It is also suggested that peripersonal space plays an important role in the body–body interactions with other people.

To study precisely this body–body dynamics, Kroczek et al. (2020) used Virtual Reality (VR) to manipulate interpersonal distance in social interactions. Participants had to interact with one of two virtual agents represented in the VR scenario. They were instructed to approach them and start interacting as soon as the agent would look up at them. The authors manipulated the distance of interaction by delaying the moment in which the virtual agent would notice the participant. They found that the closer the participant had to get to the virtual agent in order to be noticed, the “more arousing, less pleasant, and less natural” the interaction was felt. Perception of close distances was also accompanied with increased levels of skin conductance. These results are consistent with the principles of Proxemics, whereby personal space is organized in concentric areas that determine the level of ease we feel being close to another person, i.e., we can empathize with them, which is based on our level of intimacy with that person (Schiano Lomoriello et al., 2018).

Proxemics is not the only discipline that has dealt with concepts of personal distances. Construal Level Theory has also attempted to explain the relationship between social, physical, and temporal distances in terms of psychological dimensions. What is meant by psychological dimension is the level of specificity or abstraction, by which information is represented, that goes from a low-level (incidental and specific) representation of events near us to a high-level (general and prototypical) representation of farther events (Henderson et al., 2006; Trope and Liberman, 2010). The possibility of a shared mechanism for the perception of these different dimensions of distances has been corroborated by fMRI studies, showing activation of the same neural network during the processing of social and physical distances. For example, in an fMRI study, Yamakawa et al. (2009) investigated the role of the parietal cortex in analytic representations of egocentric mapping, which is employed for processing both physical and social relationships. The authors asked participants to perform two tasks. In the first task, participants had to evaluate their physical distance to neutral objects displayed on a screen. In the second task, participants were shown with two faces and had to choose the one with which they felt more compatible. Hemodynamic response was collected during both tasks and revealed a common activation in the parietal cortex. The social distance task was also linked with the activation of extended regions dedicated to social cognition processes, such as the fusiform gyri, the bilateral medial frontal cortices, the inferior frontal cortices, the insular cortices, the left basal ganglia, and the amygdala. Nevertheless, the overlap in the parietal cortex seems to confirm a common neural substrate for the evaluation of spatial and social distances, and indicates that this area is part of the network dedicated to the processing of social stimuli.

It has been argued that the parietal cortex organizes complex social information in a self-referred map of social distances, guiding our spatial behavior toward others (Abraham et al., 2008; Yamazaki et al., 2009; Parkinson and Wheatley, 2013). This supports the idea that visuospatial perception and social cognition are interconnected processes, subserved by a common substrate in the brain. The reciprocal influence of these two kinds of distances is becoming more and more evident in the literature. For example, Schiano Lomoriello et al. (2018) have demonstrated that when people are physically distant from us, we are less prone to empathize with them. In other words, the feeling of social distance or proximity is modulated by the physical distance between us and the other person. The effects are visible also the other way around, in that social inferences (e.g., categorization and stereotyping) can tweak our perception of the physical world, as demonstrated by Xiao and Bavel (2012) in their three studies on collective identity and identity threat. The authors found that threatening social situations were judged spatially closer than the non-threatening ones, reinforcing the idea of how distance perception serves the function of adjusting our behavior in relation to our social and physical environment. A final remark is on the application of the rules of physical and social distance not only to our egocentric perspective but also in the interpretation of social scenes in which more agents are interacting. The study of Zhou et al. (2019), among others, demonstrated that closer interpersonal distances, more direct interpersonal angles and more open postures, are all visual cues of ongoing interaction in a group of people. This study along with other experiments on how we interpret social scenes are described in greater detail in section 4.2 that is dedicated to the observation of multiple agents.

The studies revised in this third section confirm that our body is the arena where we enact our own and other people’s feelings, and the key to our complex social abilities. A summary of the critical studies in support of this concept is presented in Table 2. We can now finally explore in more detail how we use our vision to understand others, by describing those body indexes, such as posture and movement, that inform us on others’ psychological state. This is the aim of the next section.

**BOX 3 Definition of psychological distance.**

This concept was first proposed by Trope and Liberman in their Construal Level Theory and was defined as the level of abstraction used to represent a phenomenon based on its temporal distance. Greater distance corresponds to greater abstraction. Now the theory includes other three categories of psychological distance: spatial, social, and hypothetical. As demonstrated also in this review, these four dimensions are strongly and systemically correlated with each other. Psychological distance is inevitably egocentric, the center is the self in the present, and it serves as a measure of the value attributed to the phenomenon of interest. Closer events/agents are perceived as more important and more likely to be acted on

**Table 2:** Summary table of the studies cited in section 3.

Study	Sample	Stimuli	Design	Results and theoretical implications
*Evidences for social embodiment*				
Duclos et al. (1989)	*N* = 74 (43 females, undergraduate students)	Faces expressing sadness, anger, fear and disgust	Participants were induced to adopt expressions of fear, anger, disgust, and sadness by contracting or relaxing specific face muscles and then asked to rate their emotional state after having performed a conflicting task, designed to disguise the experiment’s goal	Although participants were naïve to the aims of the experiment, they reported the highest rating of emotion in the condition in which they were expressing that emotion, giving strength to the idea that the activation of specific sensorimotor schemas elicits those embodied feelings
Rütgen et al. (2015)	*N* = 102 (70 females, mean age = 25)	Painful stimulation delivered to the participant or to another person	The researchers induced placebo analgesia induced in half of the participants and tested all participants’ pain perception and empathy. fMRI activation and self-reported pain and empathy ratings were collected	Compared to the control group, participants with induced placebo analgesia showed reduced first-hand pain perception and reduced pain empathy, suggesting that pain empathy might be grounded in our own pain experiences
Lenzoni et al. (2020)	*N* = 66 (42 patients with myotonic dystrophy; 24 healthy controls)	FEAST-N, the subtest of the Facial Emotion Matching test, and BEAST-N, the subtest Body Emotion Matching	Emotional recognition ability was assessed in patients with myotonic dystrophy and compared with healthy controls	The clinical population performed significantly worse than the healthy controls, demonstrating a causal role of visuomotor abilities in emotion recognition
Ijzerman and Semin (2009)	*N* = 33 in Exp. 1; 52 in Exp. 2; 39 in Exp. 3	Inclusion of Other in Self (IOS) scale	Participants were asked to hold cold or hot beverages and rate the social proximity they felt with a known person of their choice using the IOS scale	Participants judged the distance between themselves and a known person as shorter when they were asked to hold a warm beverage, demonstrating an association between warmth and feelings of social proximity
Study	Sample	Stimuli	Design	Results and theoretical implications
*Evidences for overlaps between social and physical space*				
Bar-Anan et al. (2007)	*N* = 10 (6 females, undergraduate students)	Different words defining close or distant social affiliation depicted in different locations in a scenario	Participants had to indicate if the position of an item on the screen was proximal or distal, independently of the meaning of the word	Words were classified faster when psychological and spatial distances were matching, compared to when the two types of distances were incongruent
Kroczek et al. (2020)	*N* = 36 (18 females, mean age = 21.75)	Virtual agents represented in a VR scenario	Participants were instructed to approach the agents in the VR and start interacting as soon as the agent looked up at them. The interpersonal distance was varied by manipulating the distance at which agents reacted to the participant’s approach. Arousal, valence, and realism rates were collected after each interaction, on a 1–100 points scale	Closer interpersonal distances were rated as more arousing, less pleasant, and less natural than longer distances
Yamakawa et al. (2009)	*N* = 24 (4 females, age range = 19–34 years)	Two inanimate objects whose relative physical positions could be inferred by texture and lighting cues (i.e., physical distance task). Pictures of two faces (i.e., social distance task)	In the physical distance task, participants had to judge which object was closer to them. In the social distance task, participants had to choose which person they felt more compatible with. fMRI data was acquired during both tasks	Results showed that the parietal cortex was activated in both tasks, suggesting a common neural substrate for the estimation of physical and social distances
Schiano Lomoriello et al. (2018)	*N* = 34 (23 females, mean age = 23)	Pictures of faces with neutral facial expression, receiving either a painful or a neutral stimulation. All faces were presented in the upright and inverted orientation and in two physical sizes, small and big, corresponding to a perceived far and close social distance	The perceived physical distance from the stimuli was manipulated through picture sizes. Participants were asked to assess the painfulness of the stimulation applied to each face presented. EEG data was collected during the task	ERPs modulations compatible with an empathic reaction were observed only for the group exposed to face stimuli appearing to be at a close social distance from the participants, i.e., big size pictures. This reaction was absent in the group exposed to smaller stimuli corresponding to face stimuli perceived from a far social distance
Zhou et al. (2019)	*N* = 148 in total across 7 experiments (mean age = 20)	Virtual avatars placed at different positions and with different face directions	Participants had to report if the avatars in the VR environment were interacting	Results showed that closer interpersonal distances, more direct interpersonal angles and more open postures, are all visual cues of ongoing interaction in a group of people

## 4. Reuniting visual perception and social cognition: The social body In neuroscientific research

Our interaction with others is substantially mediated by the observation of their behavior. As we just described above, we understand others’ inner states by embodying their posture and expression, which elicit specific affective responses that we cognitively interpret and recognize (see subsection 3.2 for the embodied account of social cognition). In other words, it is by observing and mirroring the bodies of others that we gain insight of their inner states. The aim of this section is to provide the reader with an overview of the most recent techniques and to inspire new lines of research in visual social cognition. We report a summary of the techniques we describe in Table 3. We will distinguish between techniques that are used to examine posture, movement, and gait of individuals, from those that are used to inspect multiple agents interactions. We will end this methodological part by reporting some evidence demonstrating the social function of our vision, followed by a discussion on the importance of reintegrating the whole body in the study of emotion processing and social cognition.

**Table 3:** Summary table of the techniques presented in sections “Measures of posture, movement, and gait”, “Measures of observed social interactions”, and “ Measures of the observer”.

	Measures of posture, movement, and gait”			Technique	Measures	Studies
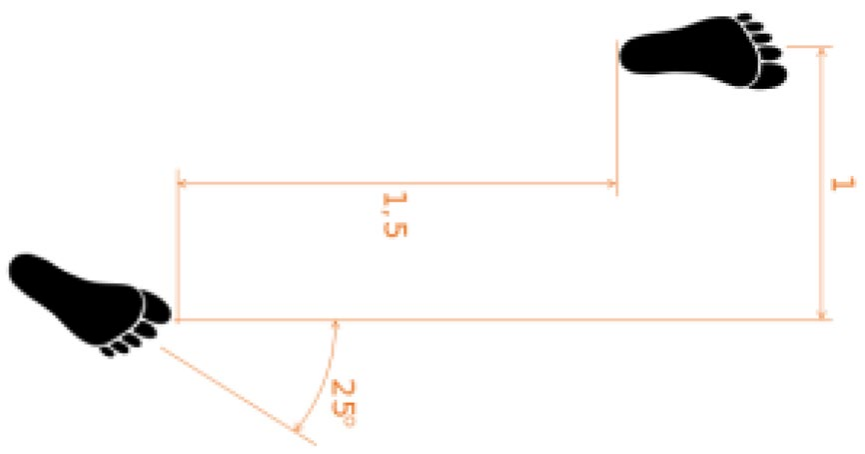 Image adapted from https://commons.wikimedia.org/wiki/File:Nachuo_sogi.svg	Stride and walk photogram analysis	Stride length and walking speed	Doumas et al. (2012); Sloman et al. (1982)
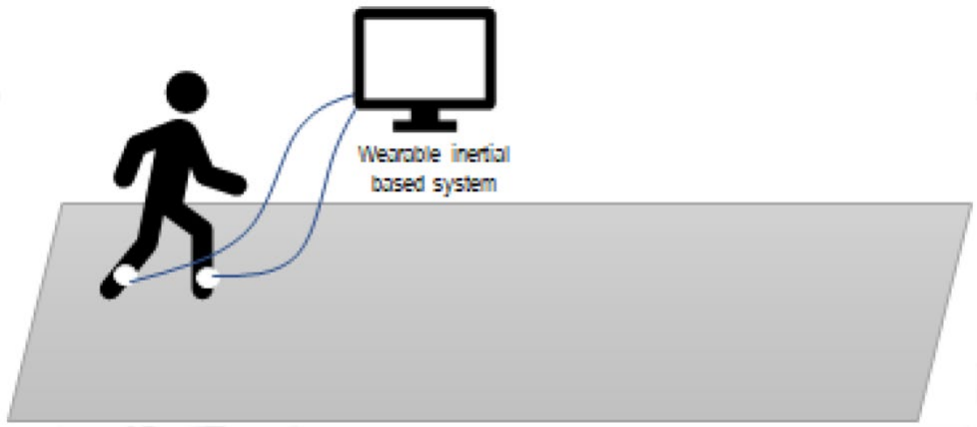	Electronic walkway	Stride length and walking speed	Lemke et al. (2000)
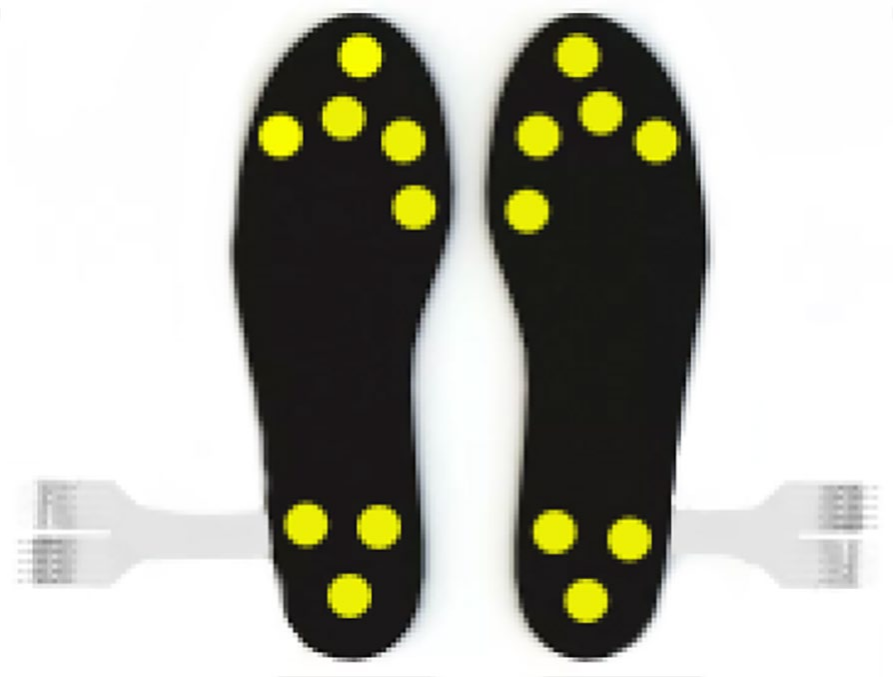	Pressure-sensitive shoe insoles	Stride length and walking speed	Hausdorff et al. (2004)
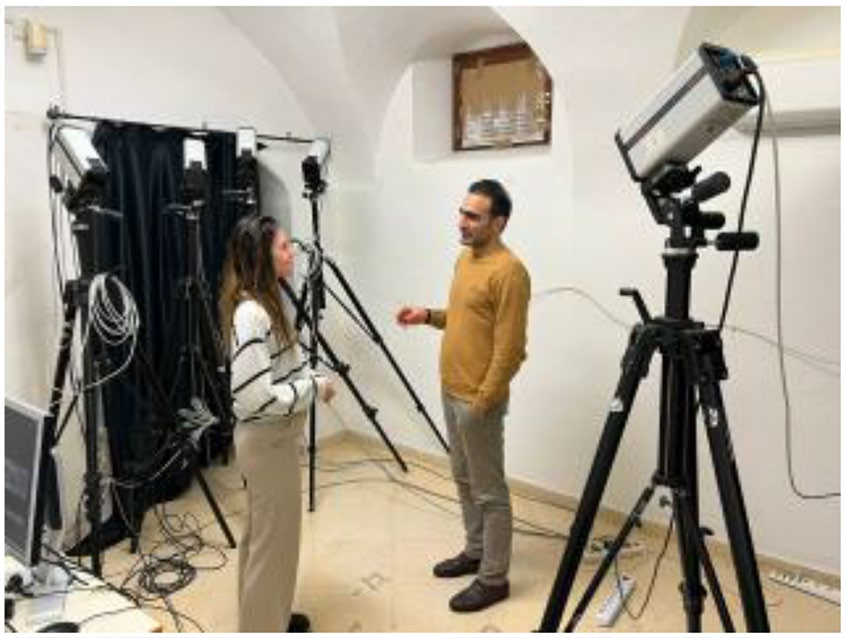	3D motion capture system (inertial motion sensors)	Diverse gait-based biomarkers	Belvederi Murri et al. (2020)
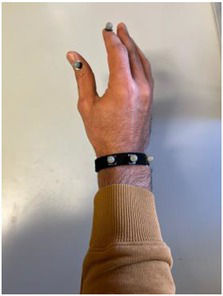	Wearable motion sensors	Gait parameters	Angelini et al. (2020)
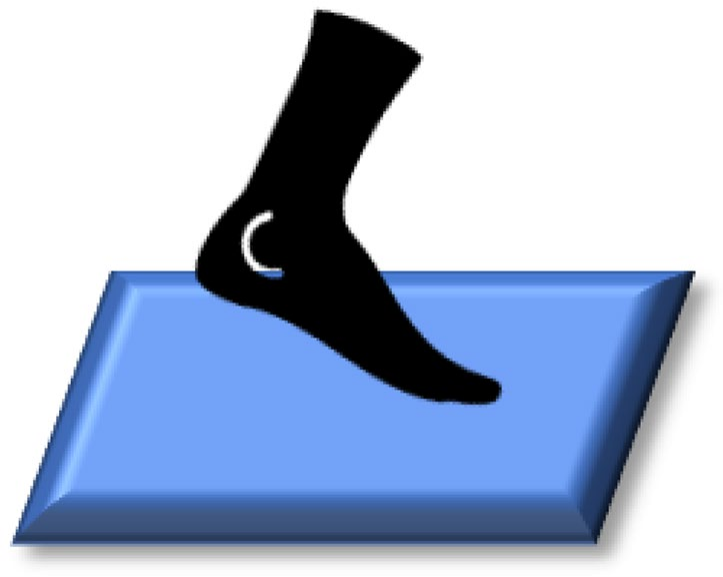 Image adapted from https://commons.wikimedia.org/wiki/File:AMTI_OPT464508_force_plate.png	Force platform	Balance	Belvederi Murri et al. (2020)
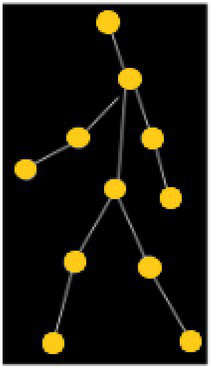	Point-light walker stimuli	Recognition of biological motion, also when it expresses emotional movements. Preference for detecting social agents facing us	Edey et al. (2017); Han et al. (2021); Miller and Saygin (2013)		Measures of observed social interactions”	Technique	Measures	Studies
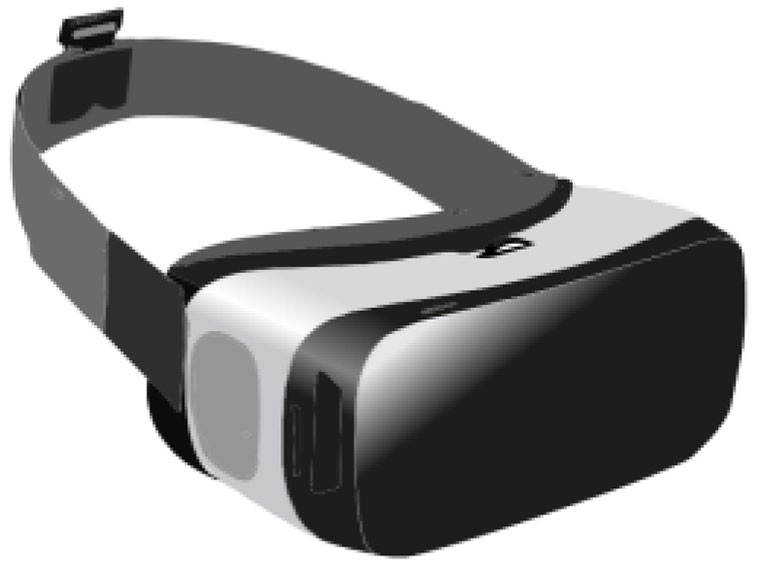 Image adapted from https://commons.wikimedia.org/wiki/File:VRHeadset.png	Virtual reality	Observing or engaging in virtual social interaction. Manipulation of body schema	Kroczek et al. (2020); Yee et al. (2009); Zhou et al. (2019)
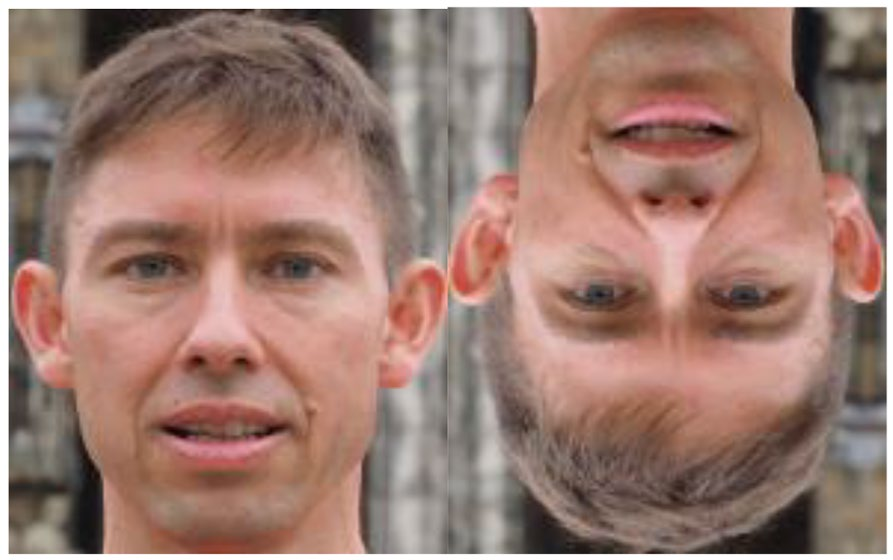 Image adapted from https://pxhere.com/en/photo/1327220	Inversion effect	Configurational representation of the image in its typical display	Papeo and Abassi (2019); Stekelenburg and Gelder (2004)
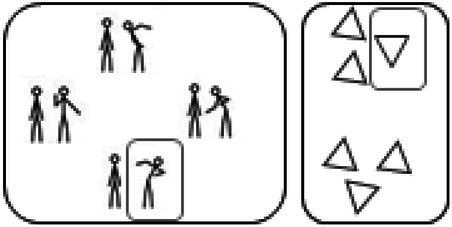	Object-inferiority effect	Faster detection of an object when represented outside a configurational figure that typically contains it	Papeo (2020)
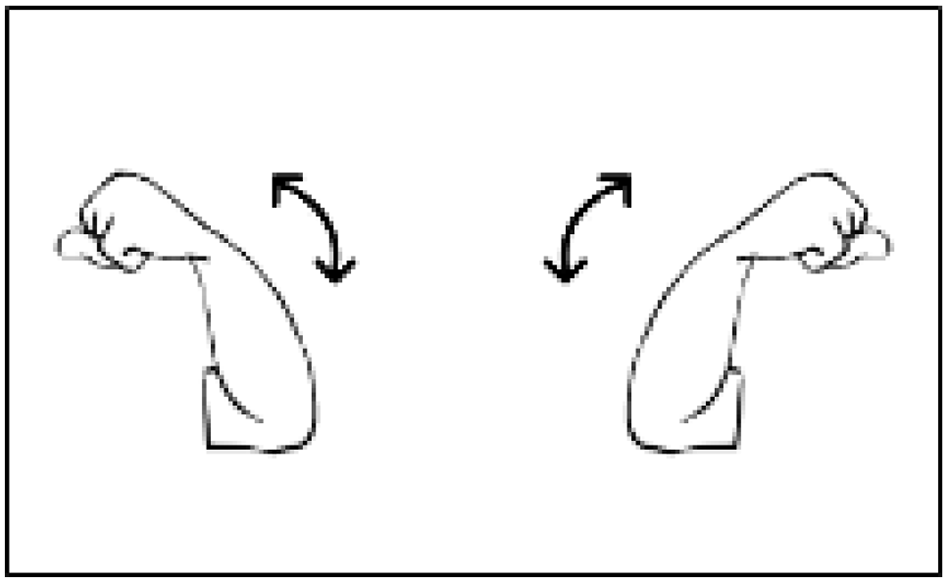	Videos of synchronous movement	Observation of social coordination as measure of social relationship	Macpherson et al. (2020)		Measures of the observer	Technique	Measures	Studies
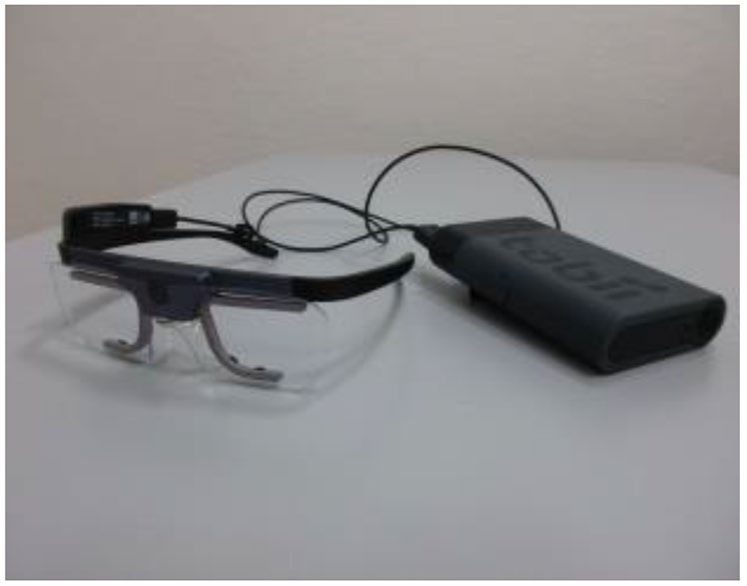	Eye-tracker (eye movements and pupillometry)	Eye position, eye movements, pupil size. Saccades, fixation duration	Kret et al. (2013)
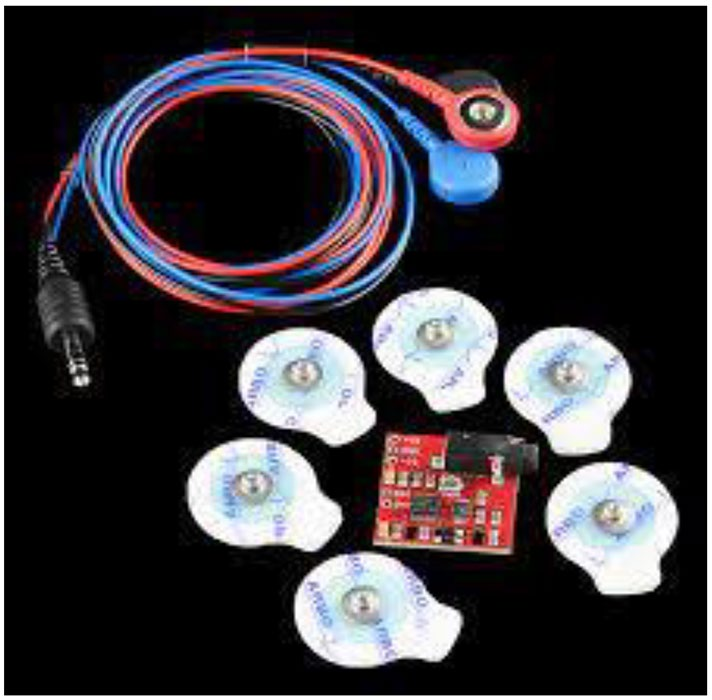 Image adapted from https://www.flickr.com/photos/sparkfun/8677911665 License CC-BY 2.0	Electromyography	Muscle activity, micro-movements	Kret et al. (2013)
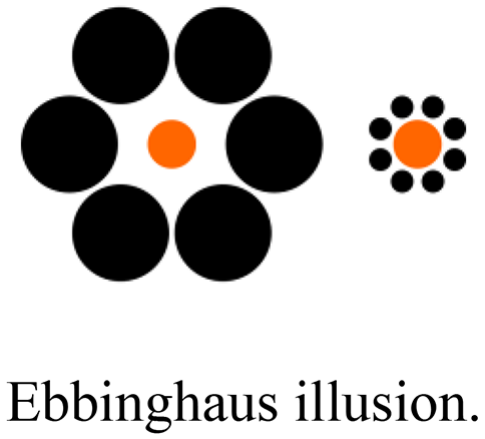 Ebbinghaus illusionImage adapted from https://www.google.com/search?q=ebbinghaus%20illusion&tbm=isch&tbs=il:cl&hl=en&sa=X&ved=0CAAQ1vwEahcKEwiYmtWH-M78AhUAAAAAHQAAAAAQAw&biw=1548&bih=937#imgrc=SCJW-hJd0NjdXM	Visual illusions	Magnitude of the illusion	Chouinard et al. (2018); King et al. (2017)
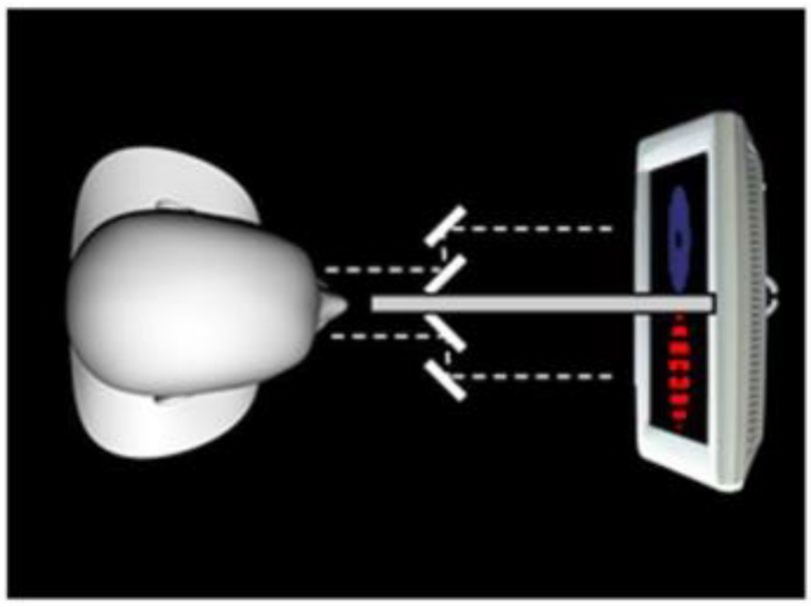 Image adapted from https://commons.wikimedia.org/wiki/File:Binocular_rivalry_Experiment.pngLicense CC-BY 4.0	Binocular rivalry	Perceptual dominance of one of two stimuli competing to reach visual awareness	Anderson et al. (2011)
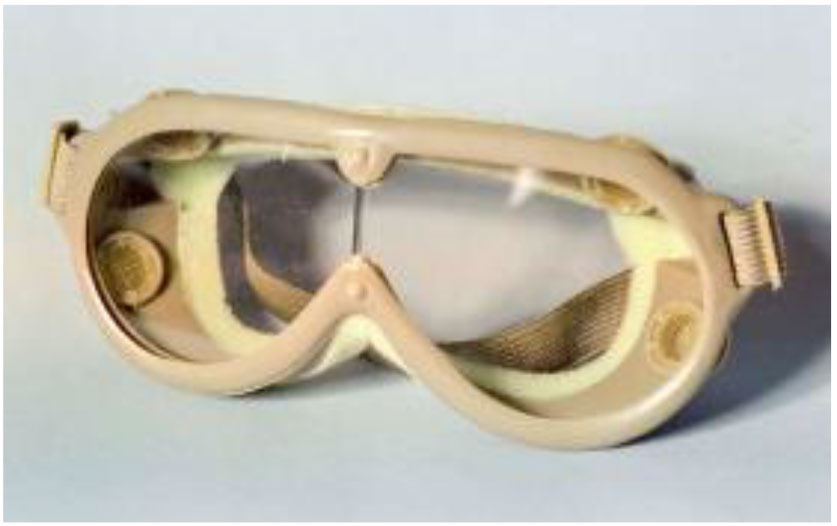 Image adapted from https://picryl.com/media/goggles-8b0197	Skewed goggles	Effects of magnification of objects	Linkenauger et al. (2010)
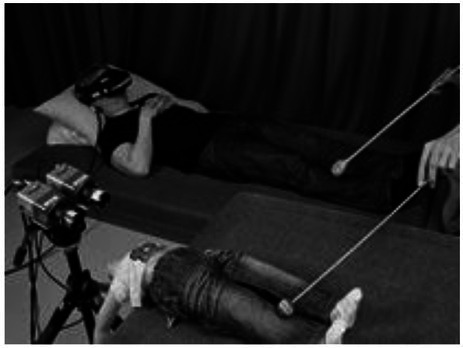 Image adapted with permission from van der Hoort et al. (2011) License CC-BY 4.0	Body-swap illusion	Influence of our body size on the perception of our physical and social environment	van der Hoort et al. (2011)

### 4.1. Measures of posture, movement, and gait

The social cues we extract from other people’s bodies are linked to their posture, movement and gait. Our emotions find expression not only by means of the facial muscles, but also in the way we position our limbs, shoulders and spine. For example, the curvature of the shoulders reflects behaviors of either closure or openness to the world, either avoidance or approaching attitude. Kinematics is another source of relevant information, and can be decomposed in different indexes: balance, movement and gait. Although the reliability of these body measures in predicting affective states is supported by an increasing number of studies, the tools and assessment methods to measure them are limited or underdeveloped in the empirical research. Here, we present a variety of instruments that can be used to quantify posture and movement.

#### 4.1.1. Posture and gait

One of the most immediate and old ways of assessing gait speed and its characteristics is by videorecording people walking and analyzing photograms of the strides. A pioneer study was conducted in the 80s by Sloman et al. (1982), in which the authors assessed the gait in adults with depression using this method. The analysis of mobility in this clinical population showed that depression is associated with specific motor symptoms, such as slower movements and worse balance compared to healthy controls (Doumas et al., 2012; Belvederi Murri et al., 2020). The use of **electronic walkways** can provide a more accurate measure of the stride length and walking speed. Lemke et al. (2000) used a combination of photogrammetry and electronic walkway and confirmed the results found by Sloman proving a reduced stride length in depressed patients. Another study on depression (Hausdorff et al., 2004) adopted **pressure-sensitive shoe insoles** to check for variability in swing time, which resulted higher in the clinical population. To obtain indexes on the posture along with the walking characteristics, the use of **3D motion capture systems** can give more detailed information about head, e.g., position and movements, upper limbs swing, back curvature. For example, studies on depressed patients have shown a correlation between the severity of the depression and the thoracic curvature, supporting the idea that a slumped position can be associated with sadness and introversion (Belvederi Murri et al., 2020). The 3D motion capture system was applied by Angelini et al. (2020) on patients with multiple sclerosis. They identified diverse gait-based biomarkers using inertial motion sensors with the goal of improving the assessment of progressive multiple sclerosis (MS). The authors examined 15 gait measures and reported longer steps and stride duration, reduced regularity and higher instability in the walk of people with MS, when compared to healthy controls. The use of **wearable sensors** for the recording of the kinematics enables the collection of data outside the lab, the identification of a variety of gait parameters and the detection of biomarkers specific to different clinical conditions.

#### 4.1.2. Balance

Balance can be assessed by means of force platforms (also known as stepping platforms) and in some cases the balance exercise performed during the execution of a working memory task can give information about how cognitive load can reduce balance skills (Doumas et al., 2012; Belvederi Murri et al., 2020). This dual task approach can be implemented also during other movement assessments, as it is effective in detecting how cognitive load influences motor skills in clinical populations.

#### 4.1.3. Observing moving bodies: Point-light walker stimuli

Obtaining gait and balance measurements can be exploited also to study our social vision: Edey et al. (2017) investigated the walking speed of participants and tested whether participants used their own kinematics as a reference to judge the affective states of point-light walker (PLW) stimuli. These visual stimuli are animations composed solely of points that have been previously attached to the joints of a moving person and extrapolated by the video recording of the scene. Following the idea that our own kinematics influences our perception of emotional movements in others, the authors manipulated the speed and posture of the artificial walker in order to elicit anger, happiness or sadness. As expected, there was a modulatory effect of the participant’s movements on the emotion recognition: people judged less intensely emotions similar to their own walking pace. In other words, participants who walked with greater speed rated high-velocity emotions (e.g., anger) as less intense relative to low-velocity emotions (e.g., sadness). This finding is in agreement with the theories of embodied social cognition reviewed above (see subsection 3.2). Point-light stimuli in motion has been used also to investigate the detection of biological motion in relation to measures of social cognition. Using this approach, it has been shown that higher scores in social cognition tests were linked to high accuracy in biological motion detection (Miller and Saygin, 2013). Moreover, PLW stimuli have been used to measure our promptness in seeing social agents facing us compared to facing away based on the perceived social relevance. The level of social relevance was manipulated by changing distance, speed and size of the PLW, based on the assumption that people perceived as nearer, faster and bigger have more social relevance than those perceived as farther, slower and smaller. Therefore, the likelihood of initiating an interaction with them increases. PLW stimuli are particularly suited to measure differences in seeing people facing toward us or away thanks to their ambiguous in-depth orientation (Han et al., 2021). Findings from these studies show once again that social factors have a clear impact on visual processes, further supporting our hypothesis of a deep link between low-level feature detection and high-level social cognition.

### 4.2. Measures of observed social interactions

Beside the interface with a single person, our social life is mainly constituted by crowded situations in which multiple agents engage complex interactions with each other. A relatively new branch of study is focusing on the perception of the relations between social entities and proposes that our interpretation of social events draws upon a configurational recognition process. Recent findings suggest a specific sensitivity of the visual system for the spatial relationship between multiple social agents, such as interpersonal distance and angle of a facing dyad (Papeo, 2020). One of the requirements for a successful social interaction is, indeed, a face-to-face position between the agents. This implies that seeing two people facing each other makes us assume an ongoing interaction (Zhou et al., 2019). Based on the data from their experiments with virtual reality (VR), Zhou et al. (2019) created a computational model of the social interaction field, which they describe as the area surrounding each of us within which we can start interacting with other people. Similarly, to a gravitational field, the social interaction field can inform us on the strength of the social interaction between two people based on their physical distance and positions in space.

#### 4.2.1. Facing dyads

We believe, akin to other authors (Papeo, 2020), that our visual system is tuned for the recognition of social interactions around us, allowing us a fast detection of social groups. The aggregation of multiple elements (individuals) in a unitary piece of visual information (group) can facilitate and fasten the representation of the crowded scene we are observing (Zhou et al., 2019). By means of the inversion effect described above, Papeo and Abassi (2019) demonstrated that facing dyads are processed as unitary perceptual objects. They found that the inversion effect was greater for the facing dyads compared to non-facing ones. To recall the definition of this phenomenon, a greater inversion effect implies greater visual sensitivity, in this case supporting the hypothesis of a visual attunement for interacting agents. It should be noted that the two bodies inversion effect is not observed for non-human or for human-object dyads. Another signature of this visual grouping is the object-inferiority effect that has been observed in the visual search through a crowd: the dyad as a whole is detected faster than the single objects within it, but only when the agents are facing each other (Papeo et al., 2019).

#### 4.2.2. Synchronicity as a measure of social relation

We extract social information about an ongoing interaction also from the observation of the agents’ movements. In this case, it is the level of interpersonal coordination that informs us about the cooperative or hostile tones of a social interaction. A higher synchronization of the dyad’s movements signals coalition rather than opposition (Macpherson et al., 2020).

Miles et al. (2009) used stick figures and sounds of footstep to simulate two people walking together with different gait patterns and found that when rhythms of walking synchrony were out of phase, these were associated with a lower level of relationship. A similar study showed that social factors, such as the skin-color, can influence the perception of **synchronous movements** (Macpherson et al., 2020).

### 4.3. Measures of the observer

After having presented numerous techniques suitable for measuring body position and movement that can be observed in one or multiple social agents, we want to offer an overview of methodologies that can be used to analyze the observer’s behavior.

For a comprehensive approach in visual social cognition, it is important to appropriately combine measures that quantify the observed social cues with measures describing the observer’s state, at a cognitive, visual and physiological level.

#### 4.3.1. Eye-trackers and other physiological indexes

Since we are concentrating on the visual aspects of social cognition, studying the eye and the way we visually scan the social scene is almost imperative. Although the most immediate way to study gaze behavior is by means of **eye-trackers**, the range of methodologies does not limit to this one. Other important indicators of social cognition - ideally to be combined with the gaze measurements - are those linked to the automatic mimicry involved in the process of emotion recognition. In this case, the focus is on the muscles and posture of the observer and the mirroring reflexes can be recorded through the application of **sensible electrodes on expression muscles**.

Kret et al. (2013) collected eye movements, pupil size, and facial muscles activity, while participants were performing an emotion discrimination task with full body and face stimuli. They used full body images from the BEAST (Bodily Expressive Action Stimulus Test, de Gelder and Van den Stock, 2011) database. The database includes stimuli representing emotions in whole-body figures, and in this experiment they were presented in association with congruent or incongruent facial expressions. Eye movements were recorded with a wearable eye-tracking device, and their analysis revealed that participants looked longer at faces than at bodies, and the same applied for happy versus angry/fearful postures, with longer fixation duration for negative body expressions compared to positive ones. Furthermore, negative emotion expressions correlated with activity in the observers’ corrugator, and happy expressions with zygomaticus’ activation. The corrugator showed more responsiveness for bodies compared to faces, whilst a reversed pattern was observed for the zygomaticus. These findings nicely dovetail with theories of embodied social cognition, supporting the preconscious activation of the expressive muscles matching the emotion observed. Other implicit indexes of emotional response are detectable by physiological measures, such as skin conductance and heart rate variability (for reviews on emotion measures see: Mauss and Robinson, 2009; Egger et al., 2019).

#### 4.3.2. Measures of visual perception

Visual illusions can provide useful insights on the interplay between high and low order processes in the perception of an image. We will describe the application of this kind of tool in more detail in the section dedicated to the clinical studies (subsection 5). Assessment of visual awareness can be provided with dichoptic stimulation (i.e., simultaneous presentation of different stimuli to the two eyes), which provokes the phenomenon of binocular rivalry, i.e., the alternation in the perception of two different images presented to each eye.

In an experiment by Anderson et al. (2011), a paradigm with binocular rivalry was used to examine the influence of the affective state of a perceiver on the visual awareness of the stimulus presented. The potential of this technique lies in the fact that the two visual inputs presented to the two visual hemispheres compete for perceptual dominance; the selective criteria are driven by top-down processes and this allows us to determine how our internal state influences visual awareness. The authors first manipulated the participants’ affective states by showing them emotional images, and subsequently asked them to perform the binocular rivalry tasks. These consisted of a neutral stimulus (e.g., a house) presented in competition with a socially relevant stimulus (facial expressions) and participants had to report what they were seeing and for how long. Results confirmed the hypothesis of the authors, for which the affective state of the viewer biases the contents of visual awareness. In fact, the social stimuli were always dominant in the image perception, and this effect was maximized when participants were asked to watch a set of stimuli inducing unpleasant emotions. These findings show how binocular rivalry can be used to explore the process of sensory selection behind our conscious experience of the world and support the role of top-down modulation on our visual perception.

#### 4.3.3. Body schema manipulations and their effects on personality

We would like to dedicate a short section also to some methodologies used to investigate the influence of our body size and appearance on our perception of the physical and social environments. In an attempt to tackle this issue, previous research has relied upon illusions, which were generated either by magnifying or minimizing the objects in the visual field, or by inducing the sensation of having a shrinked or gigantified body. In a study, Linkenauger et al. (2010) observed the rescaling effects induced by placing one’s own hand close to objects, whose size was distorted by means of skewed goggles. The presence of a personal body part canceled out the magnifying or minimizing effect of the illusion. In another study, van der Hoort et al. (2011) generated a deeper manipulation of the body schema, referred to as body-swap illusion, by touching a part of the participants’ body and showing them a video of the same tactile stimulation being performed on a mannequin of different dimensions. Although the retinal images remained identical, perceiving a different size of the body changed the estimates of size and distance of objects present in the scene (van der Hoort et al., 2011).

Lastly, virtual avatars can alter our self-representation. Yee et al. (2009) had people interact in virtual environments with avatars of different dimensions, and found that taller and attractive avatars outperformed shorter avatars in the online game “World of Warcraft.” The authors attributed the better performance to an increased self-esteem and confidence linked to the height and attractiveness of the characters, showing that an avatar’s appearance can influence a user’s behavior in an online environment. The effect was transposed also outside the virtual environment: in a second experiment, a VR session in which participants had either a tall or short avatar was followed by a face-to-face interaction during which a negotiation task was performed. It turned out that people that had embodied a tall avatar were more likely to act unfairly to gain more profit and less prone to accept transactions against their interests than participants in short avatars. These studies reveal the potential of VR as a promising technique not only for their observational scope but also as promising intervention tools in clinical settings.

### 4.4. Our eyes at the service of emotion recognition and social communication

The inextricable connection between vision and social cognition has biological plausibility: the human eye (Schutt et al., 2015). Our eyes seem to have evolved to serve the fine and complex phenomenon of human communication and the development of social skills through our gaze-following abilities. These abilities are favored by certain characteristics specific to our species: the white sclera of our eyes and the high contrast in eye and facial skin coloration. Indeed, only in humans the outline of the eyes and the position of the iris are so clearly visible, conveying information on where the others are looking (Proffitt, 2006; Tomasello et al., 2007). In a study by Tomasello et al. (2007), gaze-following behavior was studied in both primates and infants. This behavior is based on cues coming from head orientation or from eyes direction. In this study, an experimenter sat in front of the ape or the child to be tested and looked up at the ceiling in different modalities: only with the eyes while keeping the head in a frontal position, bending backward the neck and facing the ceiling with the eye closed, or with face and eyes both looking up. Results showed a preference in infants for eye direction cues independently from the head orientation, while great apes relied mostly on head direction cues, suggesting that humans are more attuned to the eyes than our closest primate relatives (the great apes) are (Tomasello et al., 2007). The unique features of the human eye probably represent the key to mechanisms of shared attention which are at the basis of the human propensity for cooperation and coordination (Shepherd, 2010).

The dominance of the visual system in human communication is evident also in the automatic and fast identification of faces and bodies even in the most complex scenarios. As anticipated in subsection 3.3, we are able to detect conspecifics and evaluate the spatial relations between them in a very rapid and preconscious way. The preconscious nature of emotion processing guided by our vision has been investigated in different studies in which patients with lesions to the primary visual cortex could still perform task of emotion discrimination, without visual awareness of the stimulus (Morris et al., 2001; Pegna et al., 2005; Tamietto and de Gelder, 2008; de Gelder, 2009). For instance, Pegna et al. (2005) collected data from one patient who became cortically blind as a consequence of two strokes that destroyed his visual cortices bilaterally. Different visual discrimination tasks showed a small capacity to discriminate emotional social stimuli (expressive faces), whilst no sensitivity was observed for different kinds of stimuli (e.g., neutral faces, animals). Similar results were obtained by Morris et al. (2001) on a patient with right hemianopia due to a left occipital lobe damage. Taken together, these findings suggest a strong connection between visual inputs and subcortical structures, aimed at providing an automatic discrimination of salient, emotional stimuli.

In summary, the human eye does not serve solely vision, but social communication and emotion processing as well (Proffitt, 2006). Once again, the distinction between cognitive and sensory processes becomes even more blurred, reinforcing those models that postulate early influences of our sociality on our perceptual systems.

### 4.5. Expressive bodies, not just faces: Reintegrating the whole body in the study of visual social cognition

Just a decade ago, only less than 5% of the experimental production had considered the inclusion of the whole body as stimuli in their design (de Gelder, 2009). By now, the situation has seen little changes: Witkower et al. (2021) argued also for the need of further investigation on how bodies convey social information. In their study on a culturally-isolated population of Nicaragua, they have shown effective recognition of bodily basic expressions of sadness, anger, and fear, in the members of this society, providing evidence for the universality of these bodily displays. Indeed, a growing body of evidence is testifying that body expressions are recognized automatically and effectively, in the same specialized manner that characterizes the innate predisposition to face perception (for a review see, Quinn and Macrae, 2011).

Behavioral and physiological data have proven that emotion recognition relies considerably on the observation of body expressions. For instance, Kret et al. (2013) presented participants with *ad-hoc* images of body emotional postures associated with congruent or incongruent facial expressions (fear or happiness). Results revealed that the recognition of the emotion expressed by the face was influenced by the emotion expressed by the body. Response time increased with incongruent stimuli, while it decreased when face and body were expressing the same feeling. Stekelenburg and Gelder (2004) examined the electrophysiological correlates of the inversion effect, a well-known phenomenon in facial perception for which people take longer to recognize faces presented upside-down compared to any other object presented in the same fashion. The effect is explained by assuming a configural representation for the identification of faces, which fastens their detection when they appear in the expected upright position but slows it down when inverted. By using EEG, the authors showed that the same ERP component, namely the N170, was evoked both by faces and bodies presented upside down, but not by pictures of inverted objects (e.g., shoes), suggesting a configural coding of bodies’ images similar to the one underlying face perception. Studies that investigated functional connectivity between brain areas active during recognition of bodily expressions (Peelen and Downing, 2005; van de Riet et al., 2009) showed the activation of the same areas that typically respond to face stimuli. These areas appeared to be only a part of the broader network involved in body stimuli processing, which also includes the supratemporal sulcus, the middle temporal/middle occipital gyrus, the superior occipital gyrus and the parieto-occipital sulcus.

These dedicated mechanisms behind body perception support the importance of recognizing bodily expressions in our everyday life.

## 5. Insights from clinical studies

Finally, lesion and clinical studies are valuable in the examination of causal relationships between social and perceptual processes. Neuromuscular diseases, for instance, can provide insights into the relation between emotion recognition in others and impaired sensorimotor skills. Such an example is the study of Lenzoni et al. (2020) on myotonic dystrophy described in section 3.2. Along with motor impairments, clinical categories in which social deficits represent the major symptomatology can be studied for investigating the connection between social cognition and perceptual anomalies. Autism spectrum disorder and schizophrenia offer the unique opportunity to examine possible links between deficits in social abilities and altered visual perception - that are typically observed in these disorders (Butler et al., 2008; King et al., 2017; Robertson and Baron-Cohen, 2017; Chung and Son, 2020).

In a review by King et al. (2017), perceptual abnormalities in schizophrenia have been revised through the analysis of studies on visual illusions and their effects on this clinical population. Perceptual illusions are widely used in vision studies, in that they allow to disentangle the mechanisms underlying visual processing. For example, the Ebbinghaus illusion (for which a target item looks smaller or bigger by effect of contextual cues) can be modulated by the effects of prior knowledge and culture on visual perception, which means that it is a distortion linked to top-down processing of the visual inputs. From the literature reviewed in King et al. (2017), it emerged that it is this kind of high-level integration that seems to be systematically altered in people with schizophrenia. In fact, they tend to show a reduced susceptibility for high-level illusions, suggesting that abnormalities in visual perception might depend on deficits in the cognitive/perceptual communication at the basis of perceptual awareness. As the same authors suggest, it would be helpful to study these processes not only in isolation but also applying converging techniques to investigate the reciprocal links between higher and lower processes with ecologically valid designs. In this way, it might be possible to explore more in depth the connections between inferential top-down aspects of visual perception and the ability of recognizing social cues from observing other people.

In a similar fashion, different perceptual styles in autism were examined in a study by Chouinard et al. (2018), in which sensory integration was investigated again by means of visual illusions. In this case, the Shepard illusion was tested in autistic and typically developing individuals while their eye movements were recorded. In contrast with the authors’ expectations, no difference was found in saccades and scene exploration between the two groups, although the clinical population experienced a weaker illusion than the healthy controls. These results can be explained by differences in high-level visual integration, instead of anomalies in earlier stages of perception (e.g., spatial exploration, saccade velocity and frequency). As for schizophrenia, the empirical data suggest that top-down inferences might be reduced in people with autism, bringing to higher objectivity in perceiving the world as it really is, which in turn leads to a diminished sensitivity to visual illusions. Once more, further research is needed in order to establish the relationship between these perceptual anomalies and deficits in higher order social cognition.

## 6. Conclusion and future directions of research

Our review strives to encourage the application of multimodal integrative approaches in cognitive, social and affective neuroscience and to inspire further research aimed at discovering the intertwined connection between social cognition and visual perception.

We highlighted the tight relationship between visual perception and social cognition. Specifically, we aimed at unveiling the role of the body as the starting point for the construction of our perception, also when it comes to social perception. Firstly, we compared research modalities that can be adopted in the exploration of these constructs, and remarked on the necessity of moving from isolationist unimodal approaches to integrative multimodal perspectives. Subsequently, we presented abundant evidence for the rooting of social cognition in bodily expressions, as defined by theories of embodied social cognition. Finally, we described a common mechanism at the basis of specific aspects of social cognition and spatial behavior: an overlapping neural network for the perception of both physical and social distances. It appears that we recruit networks dedicated to the processing of physical distances to map our social environment, strengthening the dependence of higher order social abilities on lower representational systems closer to perceptual networks.

This visual-social interface is at play also in processes of emotion recognition, as we rely on visual cues collected through the scrutiny of the other’s body. Again, the body acts as a middle ground where our vision and our social abilities can encounter.

We also described some of the body features we observe to assess the other’s state, and reviewed instruments and techniques useful in quantifying these indexes for research applications. We described different ways to measure posture, balance, and gait, as meaningful indicators of emotional states. Also the interaction among multiple agents was covered in the methodological section, by providing examples of studies that examined interpersonal distance and synchronization as a hint for understanding the quality of the relationship. Finally, we described different ways to analyze the observer’s body, such as the detection of micro-movements underlying a first stage of emotional contagion, or the visual exploration of the stimuli by means of eye-tracking devices. The body is the key for any level of reciprocal understanding, and combining the study of bodily expressions with the analysis of visual behavior can be beneficial for the development of a detailed model of social cognition.

### 6.1. Limitations

A major limitation of this review is that we were unable to extensively cover the whole literature on the topics discussed here. Due to space constraints, sometimes we failed to establish a balance between sources supporting and those opposing a particular view. In this paragraph, we would like to at least introduce the reader to the ongoing debate on what we believe to be one of the core themes of the review: the embodied account of cognition.

Probably the main criticism against embodiment theories is that they disregard any mental constructs of the perceived events. In an interesting paper by Borg (2018), the main argument against these theories relies on the absence of mental state attribution in action understanding, as we predict or explain the behavior of others by adopting what she refers to as a ‘smart behavior reading’.

According to this model, action understanding depends directly on non-mentalistic interactive embodied practices (e.g., sensitivity to physical context and bodily motions) rather than on our ability to understand and interact with others. As such, the smart behavior reading account does not take into consideration the individuality of the observed person and all the information we might have about their personality, or life circumstances, which would enable us to predict completely different outcomes of their actions. Hence, the slow, controlled and demanding characteristics required by a mentalistic interpretation of other people’s behavior are ‘deflated’ by the embodied accounts of social cognition in favor of a fast, effortless and automatic behavior reading. Nonetheless, we also believe that mental state attribution cannot be completely disregarded by models of action understanding without generating gaps/errors in interpretation, and only an integrative approach which combines both smart behavior tracking and mental state attribution would enable successful action prediction.

Another critique of the embodied accounts comes from studies by Mahon and Caramazza (2008) and Caramazza et al. (2014). Specifically, these authors argue against the explanation of previous electrophysiological research only in light of an embodied view of action understanding processes. The authors claim that the empirical evidence provided by neuroimaging research can be equally used in support of disembodied views of conceptual representations or at least they do not necessarily discard them. Motor and sensory activation during action representation can be seen as part of a cascade process that propagates through qualitatively different levels of processing. Nevertheless, Caramazza et al., also acknowledge the authenticity of sensory-motor activation during action observation or evocation, and propose instead a middle-ground theory that combines together the abstract and symbolic levels of some concepts with the more embodied instantiation of online conceptual processing (Mahon and Caramazza, 2008; Caramazza et al., 2014).

### 6.2. Future directions

This review is not the first nor the only one that points out the importance of the body in shaping cognitive processes (see for example, Harris et al., 2015). Nonetheless, it strives to have both theoretical and practical implications. The summary of different methodologies and instruments measuring body indexes and psychological responses can be a useful source of information for researchers interested in conducting studies using the paradigms described here.

For instance, physiological measures may be included in studies of visual perception to explore whether the awareness of the body to external visual stimuli precedes their conscious appraisal or vice versa, deepening our understanding of implicit and explicit processing of emotional stimuli. Moreover, recentering clinical investigations on the body indices could be particularly relevant in those syndromes characterized both by deficits in social cognition and visual perception, such as autism or schizophrenia. Critically, in these syndromes verbal communication can be severely impaired or hardly accessible. Future studies may investigate the use of the body for patterns of interactions with the external world (e.g., postures, gait) as a way to access these clinical conditions. For example, the numerous studies on synchronization as an indirect measure of relational quality could inspirate group or couple exercises aimed at eliciting cooperative behavior.

A clearer understanding of the interplay between visual perception and social cognition might also help the development of novel clinical treatments or cognitive training that takes into account perceptual alterations in order to improve social abilities. For instance, differences in visual processing in individuals with autism (e.g., less semantic-oriented, more detail-oriented) have been identified in the literature and linked to altered abilities in social cognition, such as emotion recognition. A novel training approach might aim at reinforcing global visual processing and this in turn might lead to an improvement in emotion recognition in this population.

Again, systematic investigations of physiological measures linked to the observation of others’ bodies may represent a turning point in the study of empathy and reciprocal understanding of others’ inner states. These investigations may represent an innovative way to assess empathy implicitly whilst overcoming issues associated with self-reported measures both in health participants and patients.

Finally, we hope that this review will further strengthen multi-level and multi-approach explanations of cognitive processes that attempt to promote the integration of embodiment theories with more traditional cognitivist approaches. We believe that these trends are already present in the literature, and that they will lead to a comprehensive framework and an integrated perspective on perception and cognition in the years to come.

In summary, we highlighted how the visual perception of our social and physical environments is mediated by bodily processes and expressions. The body is behind any possible interaction with our surroundings and our conspecifics. Observing other people’s bodies informs us on their psychological state and allows emotional sharing and understanding. Our body determines the way we look at the world surrounding us.

## Ethics statement

Written informed consent was obtained from the individual(s) for the publication of any potentially identifiable images or data included in this article.

## Author contributions

CD, IS, and FM contributed equally to the literature search, literature discussion, and writing of the manuscript. All authors contributed to the article and approved the submitted version.

## Conflict of interest

The authors declare that the research was conducted in the absence of any commercial or financial relationships that could be construed as a potential conflict of interest.

## Publisher’s note

All claims expressed in this article are solely those of the authors and do not necessarily represent those of their affiliated organizations, or those of the publisher, the editors and the reviewers. Any product that may be evaluated in this article, or claim that may be made by its manufacturer, is not guaranteed or endorsed by the publisher.

## References

[ref1] Abraham A. Werning M. Rakoczy H. von Cramon D. Y. Schubotz R. I. (2008). Minds, persons, and space: an fMRI investigation into the relational complexity of higher-order intentionality. Conscious. Cogn. 17, 438–450. doi: 10.1016/j.concog.2008.03.011, PMID: 18406173

[ref3] Ainley V. Apps M. A. J. Fotopoulou A. Tsakiris M. (2016). Bodily precision: a predictive coding account of individual differences in interoceptive accuracy. Philos. Trans. R. Soc. B Biol. Sci. 371:20160003. doi: 10.1098/rstb.2016.0003, PMID: 28080962 PMC5062093

[ref4] Anderson E. Siegel E. H. Barrett L. F. (2011). What you feel influences what you see: the role of affective feelings in resolving binocular rivalry. J. Exp. Soc. Psychol. 47, 856–860. doi: 10.1016/j.jesp.2011.02.009, PMID: 21789027 PMC3141576

[ref5] Angelini L. Hodgkinson W. Smith C. dd J. M. Sharrack B. Mazzà C.. (2020). Wearable sensors can reliably quantify gait alterations associated with disability in people with progressive multiple sclerosis in a clinical setting. J. Neurol. 267, 2897–2909. doi: 10.1007/s00415-020-09928-8, PMID: 32468119 PMC7501113

[ref6] Balcetis E. Dunning D. (2006). See what you want to see: motivational influences on visual perception. J. Pers. Soc. Psychol. 91, 612–625. doi: 10.1037/0022-3514.91.4.612, PMID: 17014288

[ref7] Balcetis E. Dunning D. (2009). Wishful seeing: more desired objects are seen as closer. Psychol. Sci. 21, 147–152. doi: 10.1177/0956797609356283, PMID: 20424036

[ref8] Bar-Anan Y. Liberman N. Trope Y. Algom D. (2007). Automatic processing of psychological distance: evidence from a Stroop task. J. Exp. Psychol. Gen. 136, 610–622. doi: 10.1037/0096-3445.136.4.610, PMID: 17999574 PMC3161424

[ref9] Barrett L. F. Satpute A. B. (2019). Historical pitfalls and new directions in the neuroscience of emotion. Neurosci. Lett. 693, 9–18. doi: 10.1016/j.neulet.2017.07.045, PMID: 28756189 PMC5785564

[ref10] Bastiaansen J. A. C. J. Thioux M. Keysers C. (2009). Evidence for mirror systems in emotions. Philos. Trans. R. Soc. Lond. Ser. B Biol. Sci. 364, 2391–2404. doi: 10.1098/rstb.2009.0058, PMID: 19620110 PMC2865077

[ref200] Beckes L. Coan J. A. (2011). Social Baseline Theory: The Role of Social Proximity in Emotion and Economy of Action. Soc. Personal. Psychol. Compass 5, 976–988.

[ref11] Belvederi Murri M. Triolo F. Coni A. Tacconi C. Nerozzi E. Escelsior A.. (2020). Instrumental assessment of balance and gait in depression: a systematic review. Psychiatry Res. 284:112687. doi: 10.1016/j.psychres.2019.112687, PMID: 31740213

[ref12] Berti A. Frassinetti F. (2000). When far becomes near: remapping of space by tool use. J. Cogn. Neurosci. 12, 415–420. doi: 10.1162/089892900562237, PMID: 10931768

[ref13] Bhalla M. Proffitt D. R. (1999). Visual–motor recalibration in geographical slant perception. J. Exp. Psychol. Hum. Percept. Perform. 25, 1076–1096. doi: 10.1037/0096-1523.25.4.1076, PMID: 10464946

[ref14] Blanke O. Slater M. Serino A. (2015). Behavioral, neural, and computational principles of bodily self-consciousness. Neuron 88, 145–166. doi: 10.1016/j.neuron.2015.09.029, PMID: 26447578

[ref15] Bonini L. Rotunno C. Arcuri E. Gallese V. (2022). Mirror neurons 30 years later: implications and applications. Trends Cogn. Sci. 26, 767–781. doi: 10.1016/j.tics.2022.06.003 35803832

[ref16] Borg E. (2018). On deflationary accounts of human action understanding. Rev. Philos. Psychol. 9, 503–522. doi: 10.1007/s13164-018-0386-3, PMID: 30220943 PMC6132409

[ref17] Brockmole J. R. Davoli C. C. Abrams R. A. Witt J. K. (2013). The World Within Reach: Effects of Hand Posture and Tool Use on Visual Cognition. Curr. Dir. Psychol. Sci. 22, 38–44 doi: 10.1177/0963721412465065.

[ref18] Bruner J. S. Goodman C. C. (1947). Value and need as organizing factors in perception. J. Abnorm. Soc. Psychol. 42, 33–44. doi: 10.1037/h0058484 20285707

[ref19] Butler P. D. Silverstein S. M. Dakin S. C. (2008). Visual perception and its impairment in schizophrenia. Biol. Psychiatry 64, 40–47. doi: 10.1016/j.biopsych.2008.03.023, PMID: 18549875 PMC2435292

[ref20] Caramazza A. Anzellotti S. Strnad L. Lingnau A. (2014). Embodied cognition and Mirror neurons: a critical assessment. Annu. Rev. Neurosci. 37, 1–15. doi: 10.1146/annurev-neuro-071013-013950, PMID: 25032490

[ref21] Changizi M. A. Hall W. G. (2001). Thirst modulates a perception. Perception 30, 1489–1497. doi: 10.1068/p3266 11817755

[ref22] Chouinard P. A. Royals K. A. Landry O. Sperandio I. (2018). The Shepard illusion is reduced in children with an autism Spectrum disorder because of perceptual rather than attentional mechanisms. Front. Psychol. 9:2452. doi: 10.3389/fpsyg.2018.02452, PMID: 30568622 PMC6290349

[ref23] Chung S. Son J.-W. (2020). Visual perception in autism Spectrum disorder: a review of neuroimaging studies. J. Korean Acad. Child Adolesc. Psychiatry 31, 105–120. doi: 10.5765/jkacap.200018, PMID: 32665755 PMC7350544

[ref24] Cole S. Balcetis E. Dunning D. (2013). Affective Signals of Threat Increase Perceived Proximity. Psychol. Sci. 24, 34–40. doi: 10.1177/0956797612446953, PMID: 23160204

[ref25] de Gelder B. (2009). Why bodies? Twelve reasons for including bodily expressions in affective neuroscience. Philos. Trans. R. Soc. B Biol. Sci. 364, 3475–3484. doi: 10.1098/rstb.2009.0190, PMID: 19884142 PMC2781896

[ref26] De Gelder B. Van den Stock J. (2011). The bodily expressive action stimulus test (BEAST). Construction and validation of a stimulus basis for measuring perception of whole body expression of emotions. Front. Psychol. 2:181. doi: 10.3389/fpsyg.2011.00181, PMID: 21886632 PMC3152787

[ref27] de Gelder B. Van den Stock J. Meeren H. K. M. Sinke C. B. A. Kret M. E. Tamietto M. (2010). Standing up for the body: Recent progress in uncovering the networks involved in the perception of bodies and bodily expressions. Neurosci. Biobehav. Rev. 34, 513–527. doi: 10.1016/j.neubiorev.2009.10.008, PMID: 19857515

[ref28] de Pinedo García M. (2020). Ecological psychology and Enactivism: a normative way out from ontological dilemmas. Front. Psychol. 11:1637. doi: 10.3389/fpsyg.2020.01637 32849003 PMC7406712

[ref29] Doerrfeld A. Sebanz N. Shi M. (2012). Expecting to lift a box together makes the load look lighter. Psychol. Res. 76, 467–475. doi: 10.1007/s00426-011-0398-4 22159762 PMC3383959

[ref30] Doumas M. Smolders C. Brunfaut E. Bouckaert F. Krampe R. T. (2012). Dual task performance of working memory and postural control in major depressive disorder. Neuropsychology 26, 110–118. doi: 10.1037/a0026181, PMID: 22059649

[ref31] Duclos S. E. Laird J. D. Schneider E. Sexter M. Stern L. Van Lighten O. (1989). Emotion-specific effects of facial expressions and postures on emotional experience. J. Pers. Soc. Psychol. 57, 100–108. doi: 10.1037/0022-3514.57.1.100

[ref32] Edey R. Yon D. Cook J. Dumontheil I. Press C. (2017). Our own action kinematics predict the perceived affective states of others. J. Exp. Psychol. Hum. Percept. Perform. 43, 1263–1268. doi: 10.1037/xhp0000423, PMID: 28639823

[ref33] Egger M. Ley M. Hanke S. (2019). Emotion recognition from physiological signal analysis: a review. Electron. Notes Theor. Comput. Sci. 343, 35–55. doi: 10.1016/j.entcs.2019.04.009

[ref34] Fabbri-Destro M. Rizzolatti G. (2008). Mirror neurons and Mirror Systems in Monkeys and Humans. Physiology 23, 171–179. doi: 10.1152/physiol.00004.2008 18556470

[ref35] Fairhurst M. Fairhurst K. Berna C. Tracey I. (2012). An fMRI study exploring the overlap and differences between neural representations of physical and recalled pain. PLoS One 7:e48711. doi: 10.1371/journal.pone.0048711, PMID: 23119093 PMC3485317

[ref36] Firestone C. Scholl B. J. (2016). Cognition does not affect perception: evaluating the evidence for “top-down” effects. Behav. Brain Sci. 39:e229. doi: 10.1017/S0140525X15000965 26189677

[ref37] Fodor J. A. (1983). The modularity of mind. Cambridge, Mass, London: MIT Press.

[ref38] Forkmann K. Wiech K. Sommer T. Bingel U. (2015). Reinstatement of pain-related brain activation during the recognition of neutral images previously paired with nociceptive stimuli. Pain 156, 1501–1510. doi: 10.1097/j.pain.0000000000000194, PMID: 25906345

[ref39] Friston K. (2018). Does predictive coding have a future? Nat. Neurosci. 21, 1019–1021. doi: 10.1038/s41593-018-0200-7 30038278

[ref40] Friston K. Kiebel S. (2009). Predictive coding under the free-energy principle. Philos. Trans. R. Soc. B Biol. Sci. 364, 1211–1221. doi: 10.1098/rstb.2008.0300, PMID: 19528002 PMC2666703

[ref41] Fuchs T. (2020). The circularity of the embodied mind. Front. Psychol. 11:707. doi: 10.3389/fpsyg.2020.01707, PMID: 32903365 PMC7434866

[ref42] Gallese V. (2007). Before and below ‘theory of mind’: embodied simulation and the neural correlates of social cognition. Philos. Trans. R. Soc. B Biol. Sci. 362, 659–669. doi: 10.1098/rstb.2006.2002, PMID: 17301027 PMC2346524

[ref43] Gallese V. (2009). Mirror neurons, embodied simulation, and the neural basis of social identification. Psychoanal. Dialogues 19, 519–536. doi: 10.1080/10481880903231910

[ref44] Gibson J. J. (2014). The Ecological Approach to Visual Perception: Classic Edition 1st Edn. New York: Psychology Press.

[ref45] Goldstone R. L. Barsalou L. W. (1998). Reuniting perception and conception. Cognition 65, 231–262. doi: 10.1016/S0010-0277(97)00047-4, PMID: 9557384

[ref46] Gross E. B. Proffitt D. (2013). The economy of social resources and its influence on spatial perceptions. Front. Hum. Neurosci. 7:772. doi: 10.3389/fnhum.2013.00772, PMID: 24312039 PMC3832788

[ref47] Hall E. T. (1963). A system for the notation of Proxemic behavior. Am. Anthropol. 65, 1003–1026. Available at: http://www.jstor.org/stable/668580. doi: 10.1525/aa.1963.65.5.02a00020

[ref48] Hall E. T. Birdwhistell R. L. Bock B. Bohannan P. Diebold A. R. Durbin M.. (1968). Proxemics [and comments and replies]. Curr. Anthropol. 9, 83–108. Available at: http://www.jstor.org/stable/2740724. doi: 10.1086/200975

[ref55] Hamilton A. F. de C. Kessler K. Creem-Regehr S. H. (2014). Perspective taking: building a neurocognitive framework for integrating the “social” and the “spatial.” Front. Hum. Neurosci. 8:35. doi: 10.3389/fnhum.2014.00403 24966824 PMC4052522

[ref49] Han Q. Wang Y. Jiang Y. Bao M. (2021). The relevance to social interaction modulates bistable biological-motion perception. Cognition 209:104584. doi: 10.1016/j.cognition.2021.104584, PMID: 33450439

[ref300] Harber K. D. Yeung D. Iacovelli A. (2011). Psychosocial resources, threat, and the perception of distance and height: support for the resources and perception model. Emotion 11:1080., PMID: 21707147 10.1037/a0023995

[ref50] Harris L. R. Carnevale M. J. D’Amour S. Fraser L. E. Harrar V. Hoover A. E. N.. (2015). How our body influences our perception of the world. Front. Psychol. 6:819. doi: 10.3389/fpsyg.2015.00819, PMID: 26124739 PMC4464078

[ref51] Hausdorff J. M. Peng C.-K. Goldberger A. L. Stoll A. L. (2004). Gait unsteadiness and fall risk in two affective disorders: a preliminary study. BMC Psychiatry 4:39. doi: 10.1186/1471-244X-4-39, PMID: 15563372 PMC538754

[ref52] Heft H. (2020). Ecological psychology and Enaction theory: divergent groundings. Front. Psychol. 11:991. doi: 10.3389/fpsyg.2020.00991, PMID: 32547449 PMC7271815

[ref53] Henderson M. D. Fujita K. Trope Y. Liberman N. (2006). Transcending the “here”: the effect of spatial distance on social judgment. J. Pers. Soc. Psychol. 91, 845–856. doi: 10.1037/0022-3514.91.5.845, PMID: 17059305

[ref54] Ijzerman H. Semin G. R. (2009). The thermometer of social relations: mapping social proximity on temperature. Psychol. Sci. 20, 1214–1220. doi: 10.1111/j.1467-9280.2009.02434.x 19732385

[ref56] Keysers C. Kaas J. H. Gazzola V. (2010). Somatosensation in social perception. Nat. Rev. Neurosci. 11, 417–428. doi: 10.1038/nrn2833 20445542

[ref57] Keysers C. Paracampo R. Gazzola V. (2018). What neuromodulation and lesion studies tell us about the function of the mirror neuron system and embodied cognition. Curr. Opin. Psychol. 24, 35–40. doi: 10.1016/j.copsyc.2018.04.001 29734039 PMC6173305

[ref58] King D. J. Hodgekins J. Chouinard P. A. Chouinard V.-A. Sperandio I. (2017). A review of abnormalities in the perception of visual illusions in schizophrenia. Psychon. Bull. Rev. 24, 734–751. doi: 10.3758/s13423-016-1168-5, PMID: 27730532 PMC5486866

[ref59] Kret M. E. Stekelenburg J. J. Roelofs K. de Gelder B. (2013). Perception of face and body expressions using electromyography pupillometry and gaze measures. Front. Psychol. 4:28. doi: 10.3389/fpsyg.2013.00028, PMID: 23403886 PMC3567353

[ref60] Kroczek L. O. H. Pfaller M. Lange B. Müller M. Mühlberger A. (2020). Interpersonal distance during real-time social interaction: insights from subjective experience, behavior, and physiology. Front. Psych. 11:561. doi: 10.3389/fpsyt.2020.00561, PMID: 32595544 PMC7304233

[ref61] Lamm C. Rütgen M. Wagner I. C. (2019). Imaging empathy and prosocial emotions. Neurosci. Lett. 693, 49–53. doi: 10.1016/j.neulet.2017.06.054 28668381

[ref62] Landau M. J. Meier B. P. Keefer L. A. (2010). A metaphor-enriched social cognition. Psychol. Bull. 136, 1045–1067. doi: 10.1037/a0020970, PMID: 20822208

[ref63] Lemke M. R. Wendorff T. Mieth B. Buhl K. Linnemann M. (2000). Spatiotemporal gait patterns during over ground locomotion in major depression compared with healthy controls. J. Psychiatr. Res. 34, 277–283. doi: 10.1016/S0022-3956(00)00017-0, PMID: 11104839

[ref64] Lenzoni S. Bozzoni V. Burgio F. de Gelder B. Wennberg A. Botta A.. (2020). Recognition of emotions conveyed by facial expression and body postures in myotonic dystrophy (DM). Cortex 127, 58–66. doi: 10.1016/j.cortex.2020.02.005 32169676

[ref65] Lindblom J. (2020). A radical reassessment of the body in social cognition. Front. Psychol. 11:987. doi: 10.3389/fpsyg.2020.00987, PMID: 32581915 PMC7291370

[ref66] Linkenauger S. Proffitt D. (2008). The effect of intention and bodily capabilities on the perception of size. J. Vis. 8:620. doi: 10.1167/8.6.620

[ref67] Linkenauger S. A. Ramenzoni V. Proffitt D. R. (2010). Illusory Shrinkage and Growth: Body-Based Rescaling Affects the Perception of Size. Psychol. Sci. 21, 1318–1325 doi: 10.1177/0956797610380700 20729479 PMC3302719

[ref68] Macpherson M. C. Fay N. Miles L. K. (2020). Seeing synchrony: a replication of the effects of task-irrelevant social information on perceptions of interpersonal coordination. Acta Psychol. 209:103140. doi: 10.1016/j.actpsy.2020.103140 32738451

[ref69] Maggio M. G. Piazzitta D. Andaloro A. Latella D. Sciarrone F. Casella C.. (2022). Embodied cognition in neurodegenerative disorders: what do we know so far? A narrative review focusing on the mirror neuron system and clinical applications. J. Clin. Neurosci. 98, 66–72. doi: 10.1016/j.jocn.2022.01.028 35134659

[ref70] Mahon B. Z. Caramazza A. (2008). A critical look at the embodied cognition hypothesis and a new proposal for grounding conceptual content. J. Physiol. Paris 102, 59–70. doi: 10.1016/j.jphysparis.2008.03.004, PMID: 18448316

[ref71] Mauss I. B. Robinson M. D. (2009). Measures of emotion: a review. Cogn. Emot. 23, 209–237. doi: 10.1080/02699930802204677, PMID: 19809584 PMC2756702

[ref72] Meconi F. Doro M. Schiano Lomoriello A. Mastrella G. Sessa P. (2018). Neural measures of the role of affective prosody in empathy for pain. Sci. Rep. 8:291. doi: 10.1038/s41598-017-18552-y, PMID: 29321532 PMC5762917

[ref74] Meconi F. Linde-Domingo J. S. Ferreira C. Michelmann S. Staresina B. Apperly I. A.. (2021). EEG and fMRI evidence for autobiographical memory reactivation in empathy. Hum. Brain Mapp. 42, 4448–4464. doi: 10.1002/hbm.25557, PMID: 34121270 PMC8410563

[ref75] Miles L. K. Nind L. K. Macrae C. N. (2009). The rhythm of rapport: interpersonal synchrony and social perception. J. Exp. Soc. Psychol. 45, 585–589. doi: 10.1016/j.jesp.2009.02.002

[ref76] Miller L. E. Saygin A. P. (2013). Individual differences in the perception of biological motion: links to social cognition and motor imagery. Cognition 128, 140–148. doi: 10.1016/j.cognition.2013.03.013, PMID: 23680791

[ref77] Morris J. S. DeGelder B. Weiskrantz L. Dolan R. J. (2001). Differential extrageniculostriate and amygdala responses to presentation of emotional faces in a cortically blind field. Brain 124, 1241–1252. doi: 10.1093/brain/124.6.1241 11353739

[ref78] Mukamel R. Ekstrom A. D. Kaplan J. Iacoboni M. Fried I. (2010). Single-neuron responses in humans during execution and observation of actions. Curr. Biol. 20, 750–756. doi: 10.1016/j.cub.2010.02.045, PMID: 20381353 PMC2904852

[ref400] Nakayama K. (2011). “Introduction: Vision Going Social.” in The science of social vision. Vol. 7. eds. R. B. Adams, R. B. Adams Jr, N. Ambady, K. Nakayama and S. Shimojo (Oxford university press).

[ref79] Niedenthal P. M. Barsalou L. W. Winkielman P. Krauth-Gruber S. Ric F. (2005). Embodiment in attitudes, social perception, and emotion. Personal. Soc. Psychol. Rev. 9, 184–211. doi: 10.1207/s15327957pspr0903_1, PMID: 16083360

[ref80] Niedenthal P. Wood A. (2019). Does emotion influence visual perception? Depends on how you look at it. Cogn. Emot. 33, 77–84. doi: 10.1080/02699931.2018.1561424, PMID: 30636535

[ref81] Noel J.-P. Blanke O. Serino A. (2018). From multisensory integration in peripersonal space to bodily self-consciousness: from statistical regularities to statistical inference. Ann. N. Y. Acad. Sci. 1426, 146–165. doi: 10.1111/nyas.13867, PMID: 29876922

[ref82] Oishi S. Schiller J. Gross E. B. (2013). Felt understanding and misunderstanding affect the perception of pain, slant, and distance. Soc. Psychol. Personal. Sci. 4, 259–266. doi: 10.1177/1948550612453469

[ref83] Otten M. Seth A. K. Pinto Y. (2017). A social Bayesian brain: how social knowledge can shape visual perception. Brain Cogn. 112, 69–77. doi: 10.1016/j.bandc.2016.05.002, PMID: 27221986

[ref84] Papeo L. (2020). Twos in human visual perception. Cortex 132, 473–478. doi: 10.1016/j.cortex.2020.06.005, PMID: 32698947

[ref85] Papeo L. Abassi E. (2019). Seeing social events: the visual specialization for dyadic human–human interactions. J. Exp. Psychol. Hum. Percept. Perform. 45, 877–888. doi: 10.1037/xhp0000646, PMID: 30998069

[ref86] Papeo L. Goupil N. Soto-Faraco S. (2019). Visual search for people among people. Psychol. Sci. 30, 1483–1496. doi: 10.1177/0956797619867295 31532709

[ref87] Paradiso E. Gazzola V. Keysers C. (2021). Neural mechanisms necessary for empathy-related phenomena across species. Curr. Opin. Neurobiol. 68, 107–115. doi: 10.1016/j.conb.2021.02.005, PMID: 33756399

[ref88] Parkinson C. Wheatley T. (2013). Old cortex, new contexts: re-purposing spatial perception for social cognition. Front. Hum. Neurosci. 7:645. doi: 10.3389/fnhum.2013.00645 24115928 PMC3792395

[ref89] Peelen M. V. Downing P. E. (2005). Selectivity for the human body in the fusiform gyrus. J. Neurophysiol. 93, 603–608. doi: 10.1152/jn.00513.2004 15295012

[ref90] Pegna A. J. Khateb A. Lazeyras F. Seghier M. L. (2005). Discriminating emotional faces without primary visual cortices involves the right amygdala. Nat. Neurosci. 8, 24–25. doi: 10.1038/nn1364, PMID: 15592466

[ref91] Popova Y. B. Rączaszek-Leonardi J. (2020). Enactivism and ecological psychology: the role of bodily experience in agency. Front. Psychol. 11:841. doi: 10.3389/fpsyg.2020.539841 33192782 PMC7607212

[ref92] Proffitt D. R. (2006). Embodied perception and the economy of action. Perspect. Psychol. Sci. 1, 110–122. doi: 10.1111/j.1745-6916.2006.00008.x, PMID: 26151466

[ref93] Proffitt D. Baer D. (2020). Perception: How Our Bodies Shape Our Minds Minds. St. Martin’s Press.

[ref94] Proffitt D. Linkenauger S. (2013). “Perception viewed as a phenotypic expression” in Action science: Foundations of an emerging discipline. eds. W. Prinz, M. Beisert, and A. Herwig (MIT Press), 171–197.

[ref95] Quinn K. A. Macrae C. N. (2011). The face and person perception: insights from social cognition. Br. J. Psychol. 102, 849–867. doi: 10.1111/j.2044-8295.2011.02030.x 21988388

[ref96] Rao R. P. N. Ballard D. H. (1999). Predictive coding in the visual cortex: a functional interpretation of some extra-classical receptive-field effects. Nat. Neurosci. 2, 79–87. doi: 10.1038/4580, PMID: 10195184

[ref97] Read C. Szokolszky A. (2020). Ecological psychology and Enactivism: perceptually-guided action vs. sensation-based Enaction 1. Front. Psychol. 11:270. doi: 10.3389/fpsyg.2020.01270, PMID: 32765330 PMC7381233

[ref98] Robertson C. E. Baron-Cohen S. (2017). Sensory perception in autism. Nat. Rev. Neurosci. 18, 671–684. doi: 10.1038/nrn.2017.112 28951611

[ref99] Rütgen M. Seidel E.-M. Pletti C. Riečanský I. Gartus A. Eisenegger C.. (2018). Psychopharmacological modulation of event-related potentials suggests that first-hand pain and empathy for pain rely on similar opioidergic processes. Neuropsychologia 116, 5–14. doi: 10.1016/j.neuropsychologia.2017.04.023, PMID: 28438708

[ref100] Rütgen M. Seidel E.-M. Silani G. Riečanský I. Hummer A. Windischberger C.. (2015). Placebo analgesia and its opioidergic regulation suggest that empathy for pain is grounded in self pain. Proc. Natl. Acad. Sci. 112, E5638–E5646. doi: 10.1073/pnas.1511269112 26417092 PMC4611649

[ref101] Sato A. Matsuo A. Kitazaki M. (2019). Social contingency modulates the perceived distance between self and other. Cognition 192:104006. doi: 10.1016/j.cognition.2019.06.018, PMID: 31229741

[ref102] Schiano Lomoriello A. Meconi F. Rinaldi I. Sessa P. (2018). Out of sight out of mind: perceived physical distance between the observer and someone in pain shapes Observer’s neural empathic reactions. Front. Psychol. 9:1824. doi: 10.3389/fpsyg.2018.01824, PMID: 30364280 PMC6193079

[ref103] Schnall S. Harber K. D. Stefanucci J. K. Proffitt D. R. (2008). Social support and the perception of geographical slant. J. Exp. Soc. Psychol. 44, 1246–1255. doi: 10.1016/j.jesp.2008.04.011, PMID: 22389520 PMC3291107

[ref104] Schutt R. K. Seidman L. J. Keshavan M. S. (Eds.). (2015). *Social neuroscience: Brain*. *mind*. *and society.* Harvard University Press.

[ref105] Serino A. (2019). Peripersonal space (PPS) as a multisensory interface between the individual and the environment, defining the space of the self. Neurosci. Biobehav. Rev. 99, 138–159. doi: 10.1016/j.neubiorev.2019.01.016, PMID: 30685486

[ref106] Serino A. Alsmith A. Costantini M. Mandrigin A. Tajadura-Jimenez A. Lopez C. (2013). Bodily ownership and self-location: components of bodily self-consciousness. Conscious. Cogn. 22, 1239–1252. doi: 10.1016/j.concog.2013.08.013, PMID: 24025475

[ref107] Sessa P. Meconi F. Han S. (2014). Double dissociation of neural responses supporting perceptual and cognitive components of social cognition: evidence from processing of others’ pain. Sci. Rep. 4:424. doi: 10.1038/srep07424, PMID: 25502570 PMC4262888

[ref108] Seth A. K. (2013). Interoceptive inference, emotion, and the embodied self. Trends Cogn. Sci. 17, 565–573. doi: 10.1016/j.tics.2013.09.007, PMID: 24126130

[ref109] Seth A. Suzuki K. Critchley H. (2012). An interoceptive predictive coding model of conscious presence. Front. Psychol. 2:395. doi: 10.3389/fpsyg.2011.00395, PMID: 22291673 PMC3254200

[ref110] Shepherd S. (2010). Following gaze: gaze-following behavior as a window into social cognition. Front. Integr. Neurosci. 4:5. doi: 10.3389/fnint.2010.00005, PMID: 20428494 PMC2859805

[ref111] Sloman L. Berridge M. Homatidis S. Hunter D. Duck T. (1982). Gait patterns of depressed patients and normal subjects. Am. J. Psychiatry 139, 94–97. doi: 10.1176/ajp.139.1.94, PMID: 7055284

[ref112] Stekelenburg J. Gelder B. (2004). The neural correlates of perceiving human bodies: an ERP study on the body-inversion effect. Neuroreport 15, 777–780. doi: 10.1097/00001756-200404090-00007, PMID: 15073513

[ref113] Sugovic M. Turk P. Witt J. K. (2016). Perceived distance and obesity: It’s what you weigh, not what you think. Acta Psychol. 165, 1–8. doi: 10.1016/j.actpsy.2016.01.012, PMID: 26854404

[ref114] Sun C. Chen J. Chen Y. Tang R. (2021). The influence of induced emotions on distance and size perception and on the grip scaling during grasping. Front. Psychol. 12:651885. doi: 10.3389/fpsyg.2021.651885, PMID: 34650465 PMC8507847

[ref115] Takahashi K. Meilinger T. Watanabe K. Bülthoff H. H. (2013). Psychological influences on distance estimation in a virtual reality environment. Front. Hum. Neurosci. 7:580. doi: 10.3389/fnhum.2013.00580, PMID: 24065905 PMC3776303

[ref116] Tamietto M. de Gelder B. (2008). Affective blindsight in the intact brain: neural interhemispheric summation for unseen fearful expressions. Neuropsychologia 46, 820–828. doi: 10.1016/j.neuropsychologia.2007.11.002, PMID: 18160081

[ref117] Teneggi C. Canzoneri E. di Pellegrino G. Serino A. (2013). Social modulation of peripersonal space boundaries. Curr. Biol. 23, 406–411. doi: 10.1016/j.cub.2013.01.043 23394831

[ref118] Tomasello M. Hare B. Lehmann H. Call J. (2007). Reliance on head versus eyes in the gaze following of great apes and human infants: the cooperative eye hypothesis. J. Hum. Evol. 52, 314–320. doi: 10.1016/j.jhevol.2006.10.001, PMID: 17140637

[ref119] Trope Y. Liberman N. (2010). Construal-level theory of psychological distance. Psychol. Rev. 117, 440–463. doi: 10.1037/a0018963, PMID: 20438233 PMC3152826

[ref120] Valenti J. J. Firestone C. (2019). Finding the “odd one out”: memory color effects and the logic of appearance. Cognition 191:103934. doi: 10.1016/j.cognition.2019.04.003, PMID: 31382106

[ref121] van de Riet W. A. C. Grezes J. de Gelder B. (2009). Specific and common brain regions involved in the perception of faces and bodies and the representation of their emotional expressions. Soc. Neurosci. 4, 101–120. doi: 10.1080/17470910701865367, PMID: 19255912

[ref122] van der Hoort B. Guterstam A. Ehrsson H. H. (2011). Being Barbie: the size of one’s own body determines the perceived size of the world. PloS One 6:e20195. doi: 10.1371/journal.pone.0020195, PMID: 21633503 PMC3102093

[ref500] Varela F. J. Thompson E. Rosch E. (1991). The embodied mind: Cognitive science and human experience. Cambridge, MA, US: The MIT Press., PMID: 33756399

[ref123] Witkower Z. Hill A. K. Koster J. Tracy J. L. (2021). Beyond face value: evidence for the universality of bodily expressions of emotion. Affect. Sci. 2, 221–229. doi: 10.1007/s42761-021-00052-y 36059900 PMC9382937

[ref124] Xiao Y. J. Bavel J. J. V. (2012). See your friends close and your enemies closer. Personal. Soc. Psychol. Bull. 38, 959–972. doi: 10.1177/0146167212442228 22510363

[ref125] Yamakawa Y. Kanai R. Matsumura M. Naito E. (2009). Social distance evaluation in human parietal cortex. PLoS One 4:e4360. doi: 10.1371/journal.pone.0004360, PMID: 19204791 PMC2635936

[ref126] Yamazaki Y. Hashimoto T. Iriki A. (2009). The posterior parietal cortex and non-spatial cognition. Biol. Rep. 1:74. doi: 10.3410/B1-74, PMID: 20948614 PMC2948259

[ref127] Yee N. Bailenson J. N. Ducheneaut N. (2009). The proteus effect: implications of transformed digital self-representation on online and offline behavior. Commun. Res. 36, 285–312. doi: 10.1177/0093650208330254

[ref128] Zadra J. R. Weltman A. L. Proffitt D. R. (2016). Walkable distances are bioenergetically scaled. J. Exp. Psychol. Hum. Percept. Perform. 42, 39–51. doi: 10.1037/xhp0000107, PMID: 26301887

[ref129] Zaki J. Ochsner K. N. (2012). The neuroscience of empathy: progress, pitfalls and promise. Nat. Neurosci. 15, 675–680. doi: 10.1038/nn.3085, PMID: 22504346

[ref130] Zhou C. Han M. Liang Q. Hu Y.-F. Kuai S.-G. (2019). A social interaction field model accurately identifies static and dynamic social groupings. Nat. Hum. Behav. 3, 847–855. doi: 10.1038/s41562-019-0618-2, PMID: 31182793

